# Long Non-Coding RNAs Differentially Expressed between Normal versus Primary Breast Tumor Tissues Disclose Converse Changes to Breast Cancer-Related Protein-Coding Genes

**DOI:** 10.1371/journal.pone.0106076

**Published:** 2014-09-29

**Authors:** Kristin Reiche, Katharina Kasack, Stephan Schreiber, Torben Lüders, Eldri U. Due, Bjørn Naume, Margit Riis, Vessela N. Kristensen, Friedemann Horn, Anne-Lise Børresen-Dale, Jörg Hackermüller, Lars O. Baumbusch

**Affiliations:** 1 Young Investigators Group Bioinformatics and Transcriptomics, Department Proteomics, Helmholtz Centre for Environmental Research – UFZ, Leipzig, Germany, and Department for Computer Science, University of Leipzig, Leipzig, Germany; 2 RNomics Group, Department Diagnostics, Fraunhofer Institute for Cell Therapy and Immunology – IZI, Leipzig, Germany; 3 LIFE Interdisciplinary Research Cluster, University of Leipzig, Leipzig, Germany; 4 Clinical Molecular Biology (EpiGen), Akershus University Hospital, Lørenskog, Norway; 5 K.G. Jebsen Center for Breast Cancer Research, Institute for Clinical Medicine, Faculty of Medicine, University of Oslo, Oslo, Norway; 6 Department of Genetics, Institute for Cancer Research, Oslo University Hospital Radiumhospitalet, Oslo, Norway; 7 Department of Oncology, Oslo University Hospital Radiumhospitalet, Oslo, Norway; 8 Department of Breast and Endocrine Surgery, Oslo University Hospital, Ullevål, Norway; 9 Institute for Clinical Immunology, University of Leipzig, Leipzig, Germany; 10 Department of Pediatric Research, Women and Children's Division, Oslo University Hospital Rikshospitalet, Oslo, Norway; University of Alabama at Birmingham, United States of America

## Abstract

Breast cancer, the second leading cause of cancer death in women, is a highly heterogeneous disease, characterized by distinct genomic and transcriptomic profiles. Transcriptome analyses prevalently assessed protein-coding genes; however, the majority of the mammalian genome is expressed in numerous non-coding transcripts. Emerging evidence supports that many of these non-coding RNAs are specifically expressed during development, tumorigenesis, and metastasis. The focus of this study was to investigate the expression features and molecular characteristics of long non-coding RNAs (lncRNAs) in breast cancer. We investigated 26 breast tumor and 5 normal tissue samples utilizing a custom expression microarray enclosing probes for mRNAs as well as novel and previously identified lncRNAs. We identified more than 19,000 unique regions significantly differentially expressed between normal versus breast tumor tissue, half of these regions were non-coding without any evidence for functional open reading frames or sequence similarity to known proteins. The identified non-coding regions were primarily located in introns (53%) or in the intergenic space (33%), frequently orientated in antisense-direction of protein-coding genes (14%), and commonly distributed at promoter-, transcription factor binding-, or enhancer-sites. Analyzing the most diverse mRNA breast cancer subtypes Basal-like versus Luminal A and B resulted in 3,025 significantly differentially expressed unique loci, including 682 (23%) for non-coding transcripts. A notable number of differentially expressed protein-coding genes displayed non-synonymous expression changes compared to their nearest differentially expressed lncRNA, including an antisense lncRNA strongly anticorrelated to the mRNA coding for histone deacetylase 3 (HDAC3), which was investigated in more detail. Previously identified chromatin-associated lncRNAs (CARs) were predominantly downregulated in breast tumor samples, including CARs located in the protein-coding genes for CALD1, FTX, and HNRNPH1. In conclusion, a number of differentially expressed lncRNAs have been identified with relation to cancer-related protein-coding genes.

## Introduction

Breast cancer is a highly heterogeneous disease with the highest cancer incidence rate and the second highest mortality rate of cancer diseases among women [1]. Tumors of breast cancer patients exhibit substantial variations in treatment response, relapse, and survival rate. Distinct mRNA expression signatures discriminate breast cancer subtypes with different clinical implications [2]–[4]. This characterization of the intrinsic molecular subtypes is solely based on protein-coding genes. However, only 1.5–2% of mammalian genomic sequences codes for proteins and it has been shown that mammalian genomes are pervasively transcribed comprising large numbers of non-coding RNAs (ncRNAs) [5]–[7]. Already 50 years ago Jacob and Monod proposed a system of double genetic control of protein synthesis in bacteria, based on structural (protein-coding) and regulatory (non-protein-coding) genes [8]. Lately, this historic view of gene regulation has achieved new attraction by the observation that many of the ncRNAs are specifically expressed depending on cell type, tissue, and developmental timing. NcRNAs are rather arbitrarily classified into two major groups, represented by short RNAs (less than 200 bp in length, including the miRNAs) and by long non-coding RNAs (lncRNAs, represented with sequence lengths of 200 bp and above). It becomes increasingly apparent that lncRNAs play an important role in central cellular processes, ranging from transcriptional and post-transcriptional regulation to the control of cellular structure integrity, subcellular localization, and epigenetic modifications [9]–[12]. LncRNAs influence transcription in either an enhancer-like fashion by rearranging chromatin via chromosomal looping [13], [14], by guiding transcription factors to their target genes [15], or by preventing the binding of transcription factors [16]. LncRNAs also affect post-transcriptional regulation by acting as miRNA sponges [17], [18] or by controlling pre-mRNA splicing, as reported for the highly abundant lncRNA MALAT1/NEAT2 [19], [20]. Further, epigenetic regulation of the cell is mediated by lncRNAs recruiting chromatin modifying complexes to specific genomic regions located either at distant or at proximal sites. The recently detected class of large intergenic non-coding RNAs (lincRNAs) comprises instances of lncRNAs modulating chromatin status in *trans* [21]–[25], whereas examples of natural antisense transcripts (NATs) and of intergenic chromatin-associated lncRNAs regulate chromatin status in *cis* [26], [27].

Several studies (e.g. [28], [29]) have illustrated that miRNAs are involved in the development and progression of breast cancer; however, detailed characterization of the impact of lncRNAs on the transition of normal to breast cancer tissue remains unknown. The emerging number of lncRNAs associated with processes that are critical for survival suggests a possible role of lncRNAs in oncogenic and tumor suppressor pathways [19], [30]–[33]. In breast cancer, several individual lncRNAs have been presented with direct influence on the cancer developmental process [34]–[37]. *HOTAIR* represents one of the most prominent examples of *trans*-regulatory lncRNAs in cancer, identified as a powerful predictor of eventual metastasis and survival [25]. Further examples of lncRNAs involved in breast cancer are the chemoresistance-related CCAT2 [38], the oncogenic H19 [39], and the tumor suppressor GAS5 [40].

Beyond individual examples of lncRNAs associated with breast cancer, the transformation of normal to tumor tissue involves dramatic changes in the genome and the transcriptome involving the deregulation of numerous lncRNAs [31], [36]. A recent transcriptome study depicted an altered distribution of sense- and antisense transcription between normal and neoplastic breast tissues [41]. Deep sequencing of lobular in-situ carcinoma further revealed that a substantial fraction of non-coding regions is transcribed in primary breast cancer [42]. LncRNAs located in the *HOX* locus display significant expression variation between normal breast epithelia versus primary and metastatic breast cancers [25]. However, none of the mentioned studies explored lncRNA differential expression variation in samples with defined molecular subtypes in comparison to the expression levels in normal breast tissue.

Here, we investigated the expression patterns of lncRNAs and mRNAs of 26 breast tumors – distributed equally between the five molecular subtypes Luminal A, Luminal B, ERBB2, Basal-like, and Normal-like – and 5 normal breast tissue samples. We applied a custom expression microarray interrogating previously identified lncRNAs regulated in tumor-relevant pathways [43], lncRNAs from public databases, and mRNAs. The focus of this study was to investigate in breast cancer the molecular characteristics and further the potential regulatory relations of lncRNAs on protein-coding genes to receive a more profound understanding of the multifaceted appearance of the disturbed processes in tumor development and progression.

## Results

### Investigating transcriptional characteristics of breast tumor patient samples and normal tissue

A custom expression microarray (GEO accession number GPL13648) was used to analyze the expression patterns of protein-coding and non-coding transcripts in total RNA from 26 well characterized breast tumor samples [44] and 5 normal breast tissue samples from breast reduction operations (Table S1). Tumor tissue samples were selected to distribute equally between the five well-established mRNA subtypes – Luminal A, Luminal B, ERBB2, Basal-like, and Normal-like - based on the PAM50 molecular classifier [2], [45]. We are aware that the samples used for our analysis include samples with heterogeneous tissue composition. Nonetheless, we observed a widespread downregulation of tumor suppressors in breast cancer tissue samples versus normal samples and an upregulation of oncogenic RNAs in tumor, both at the coding and non-coding level (Tables S2 and S3). Moreover, we did not detect any enrichment for adipocyte-specific pathways (KEGG pathway identifiers 00061, 00062, 00071, 00532, 00533, 00534, 01040, 04975) suggesting that differential expression of novel or functionally unannotated non-coding transcripts relates mainly to the transition to tumor. We used Fisher's exact test to assess whether the observed overlap of significantly regulated transcripts with genomic annotation would have been detected by using randomly chosen probes from the array. Odds ratios were computed between the relative overlap of significantly differentially expressed probes (DE-probes) and the annotations over the relative overlap of the annotations and all probes contained on the microarray. We report the observed odds ratios, their 95% confidence intervals and p-values, accordingly.

Differentially expressed transcripts that mapped to intergenic or intronic space were considered as *bona fide* non-coding (subsequently referred as non-coding), if they did not exhibit any evidence for encoding functional open reading frames, as predicted by RNAcode (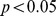) [46], nor any amino-acid sequence similarity to known proteins as annotated in the RefSeq database from March 7, 2012 (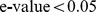) [47]. Additionally, we obtained a separate set of non-coding transcripts antisense to exons of known protein-coding genes; however, without any bioinformatic evidence for a functional open reading frame on the reading strand of the probe.

### Non-coding transcription changed drastically between normal and tumor samples, independent of copy number changes

**Figure 1:**
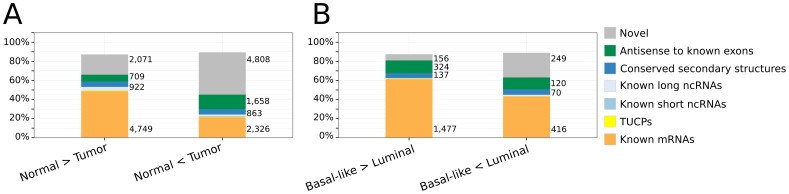
Differential expression analysis. The expression patterns of mRNA-probes and non-coding probes of 26 breast tumors and 5 normal breast tissues were investigated using the custom microarray. (**A.**) Fraction of unique genomic loci significantly differentially expressed (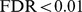) between normal and tumor samples located completely in exons of protein-coding genes (Gencode v12), in exons of known lncRNAs (lincRNAs, Gencode v12 lncRNAs, lncRNAs as annotated in lncRNAdb [51], and lncRNAs contained in chromatin [27]), in exons of transcripts of uncertain coding potential (TUCPs [23]), in exons of short RNAs (UCSC sno/miRNA track), in genomic loci with conserved secondary structure motifs (Evofold [59], RNAz [58], [97] and SISSIz [53]), in antisense-direction to known exons (Gencode v12), or in novel genomic regions. (**B.**) Fraction of unique genomic loci significantly differentially expressed (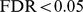) between Basal-like and Luminal tumors and located in genomic annotations as described for panel A. Numbers beside bars denote absolute number of unique DE-loci.

Our analysis revealed 20,605 probes corresponding to 19,245 unique loci (hg19) significantly differentially expressed (DE) between normal and tumor samples (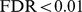), reflecting major differences in the transcriptional landscape of breast cancer in contrast to normal tissue (Figure 1A, Figure S4). Of these regulated genomic loci mapped 7075 (36%) to exons of known protein-coding genes (Gencode v12), 3882 (20%) were located in intergenic space, and 6047 (31%) in introns of protein-coding genes.

**Figure 2:**
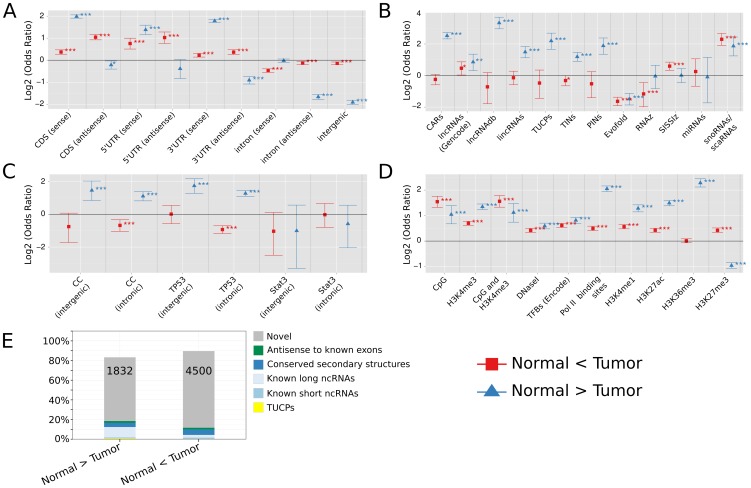
DE-probe overlap with genomic annotation (Normal versus Tumor). **A.–D.**: Number of DE-probes significantly differentially expressed between normal and tumor samples (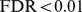) and mapping to different genomic annotations. Log2 transformed odds ratios and their 95% confidence interval for the respective annotation dataset are shown. Odds ratios of observed versus expected probe overlaps were calculated and tested by Fisher's exact test for significant enrichment or depletion, with *** indicating 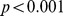, ** 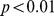, and * 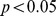, respectively. Results are shown (**A.**) for DE-probes located in annotated protein coding genes versus intergenic space based on Gencode release v12, (**B.–D.**) for intergenic or intronic non-coding DE-probes either located in several classes of known and predicted ncRNAs (B.), in non-coding transcripts regulated during cell cycle (CC), upon TP53 or Stat3 induction [43] (C.), or in regulatory sites (D.). (**E.**) Fraction of unique non-coding DE-loci in exons of known short and long ncRNAs, in genomic sites with conserved secondary structures, in antisense-direction to known non-coding exons (Gencode v12), or in novel sites. Numbers denote absolute number of DE-loci located in novel sites. For detailed output of Fisher's exact tests see Table S4, and Table S7 for detailed description of annotation datasets.

DE-probes downregulated in tumor were highest enriched, according to Fisher's exact test, in exons of protein-coding genes (Figure 2A, and Table S4), many of these are known to act as tumor suppressors [48] (odds ratio 5.2 fold, 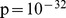, data not shown). DE-probes upregulated in tumor appeared to represent primarily novel transcripts (Figure 1A), which was also reflected by a comparably lower enrichment in exons of protein-coding genes (Figure 2A, Table S4). Many tumor-upregulated DE-probes were found antisense to exons of protein-coding genes (Figure 1A, Figure 2A), suggesting that an abundant repertoire of antisense transcripts is active in breast cancer tissue but not in normal breast tissue.

An impressive number of 8591 (82.9%) DE-probes that mapped to 8282 unique genomic loci in intergenic or intronic space were non-coding, i.e. without any bioinformatic evidence for functional open reading frames or sequence similarity to known proteins. Non-coding DE-probes located antisense to exons of known protein-coding genes mapped to 1365 unique genomic loci.

It ought to be expected that at least some of the observed expression changes of non-coding loci might be driven by alterations at the DNA level, since it has been shown that 12% of the total variation of mRNA expression in breast cancer may be explained by copy number variation [49], [50]. Consequently, we evaluated the influence of DNA copy number variation on in-*cis* lncRNA expression changes. Correlation analysis of the 26 tumor samples utilizing data from 109k Illumina SNP array did not detect any in-*cis* correlation between copy number and lncRNA expression (absolute Spearman's correlation coefficients 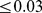) (Figure S1B). Following *Pollack et al. 2002*, we applied a linear regression model to estimate that the expression variation of 6% of the non-coding probes was explained by copy number variation [50]. These results indicate that the contribution of copy number changes to the observed variation in the lncRNA expression is marginal and that the differential expression of lncRNAs in breast cancer has more complex reasons than a trivial consequence of genomic aberrations.

### Non-coding transcription was altered within tumor samples

To investigate the biological variation of lncRNAs within tumor samples independent of known mRNA subtypes, we applied unsupervised hierarchical clustering. Uncertainty of derived clusters was assessed by random sampling with replacement (bootstrapping with 10,000 iterations). Hierarchical clustering based on probes mapping to protein-coding exons largely reproduced the known mRNA subtypes and reflected TP53 status, while hierarchical clustering based on non-coding probes revealed a different pattern (Figure S3). However, due to the small number of patient samples and the associated limited discriminative power of unsupervised clustering methods, this discrepancy should be interpreted with caution.

An F-test was employed to assess, if the mean expression of a probe is equal for all five mRNA-based subtypes in our sample set. We identified 3175 probes that were significantly differentially expressed between any subtype (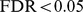). We chose a less stringent false discovery rate to account for smaller sample sizes in each group, 382 of these probes mapped to non-coding regions of the human genome with a distinct expression pattern for the Basal-like tumors (Figure S2).

**Figure 3:**
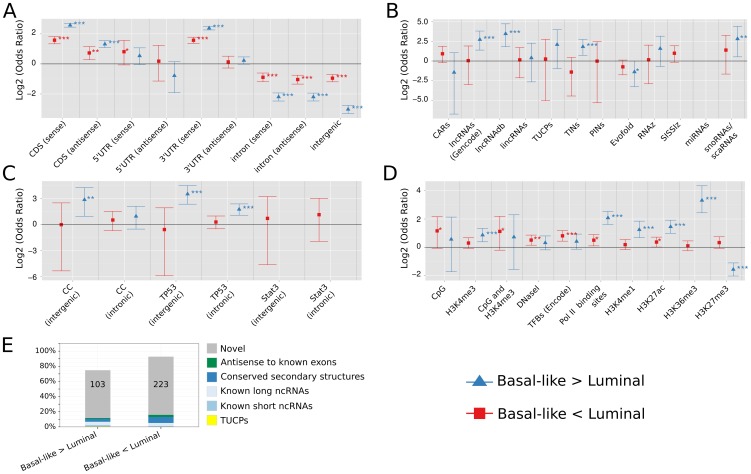
DE-probe overlap with genomic annotation (Basal-like versus Luminal A and B tumors). **A.–D.**: Number of DE-probes significantly differentially expressed between Basal-like and Luminal A and B tumors (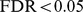) and mapping to different genomic annotations. Log2 transformed odds ratios and their 95% confidence interval for the respective annotation dataset are shown. Odds ratios of observed versus expected probe overlaps were calculated and tested by Fisher's exact test for significant enrichment or depletion, with *** indicating 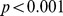, ** 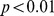, and * 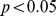, respectively. Missing error bars denote no DE-probes overlapped with according annotation. Results are shown (**A.**) for DE-probes located in annotated protein coding genes versus intergenic space based on Gencode release v12, (**B.–D.**) for intergenic or intronic non-coding DE-probes either located in several classes of known and predicted ncRNAs (B.), in non-coding transcripts regulated during cell cycle (CC), upon TP53 or Stat3 induction [43] (C.), or in regulatory sites (D.). (**E.**) Fraction of unique non-coding DE-loci in exons of known short and long ncRNAs, in genomic sites with conserved secondary structures, in antisense-direction to known non-coding exons (Gencode v12), or in novel sites. Numbers denote absolute number of DE-loci located in novel sites. For detailed output of Fisher's exact tests see Table S4, and Table S7 for detailed description of annotation datasets.

In breast cancer, the most extreme diverging mRNA subgroups are Luminal A and B versus the Basal-like subtype, different in hormon status, prognosis, and survival rate. Our analysis focused on the comparison of these outermost subgroups and we identified 3025 unique genomic regions significantly differentially expressed (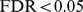), of which 682 were non-coding. The majority (60%) of unique loci that were upregulated in Basal-like tumors corresponded to exons of protein-coding genes (Figure 1B). Of the remaining, 324 (15%) loci were identified in antisense direction of known exons (275 to exons of known protein-coding genes), 134 (6%) corresponded to known lncRNAs or predicted lncRNAs with conserved secondary structures, and 156 (7%) loci were novel. In contrast, only 45% (416) of the unique genomic loci upregulated in the Luminal A and B tumors mapped to coding genes but approximately one third (249) mapped to novel loci. Differential expression of antisense transcripts was higher than expected from the composition of the custom microarray (Figure 3A).

### Differentially expressed non-coding transcripts were mostly novel

We observed that up to 80% of non-coding DE-loci, either with significant expression changes between normal and tumor or Basal-like and Luminal A and B samples, were novel. They were neither antisense to known exons, nor located in known or predicted short or long ncRNAs (Figures 2E and 3E). Some of these novel regions corresponded to non-coding transcripts, previously identified to be regulated by cancer-related pathways, like mitotic cell cycle and TP53 mediated apoptosis [43]. We observed an overrepresentation of those within the set of non-coding transcripts downregulated in breast cancer tissue (Figure 2C) or upregulated in Basal-like tumor samples (Figure 3C).

Apart from transcriptional changes in novel sites, we found also significant regulation of known lncRNAs. Considering all non-coding DE-probes downregulated in tumor samples, we detected an enrichment for lincRNAs [23], lncRNAs as annotated in Gencode v12, chromatin-associated lncRNAs (CARs [27]), manually curated lncRNAs (lncRNAdb [51]), transcripts originating from introns of protein-coding genes [52], transcripts of uncertain coding potential (TUCPs [23]), and small RNAs (Figure 2B, Table S4). Non-coding DE-probes upregulated in tumor displayed significant enrichments for lncRNAs as annotated in Gencode v12, for small RNAs, and for loci of conserved secondary structures (SISSIz [53]).

Within the tumor samples, DE-probes upregulated in Basal-like tumors showed significant enrichment for known lncRNAs (Gencode v12, lncRNAdb [51]), for non-coding RNAs transcribed from introns [52], and for small RNAs (Figure 3B and Table S4).

### Chromatin-associated lncRNAs were mostly downregulated in tumor samples

One of the highest scored enrichments of non-coding DE-probes, which were significantly differentially expressed between normal and tumor tissue, was observed for lncRNAs previously detected to be contained in chromatin of human fibroblast cells [27]. A total of 88 chromatin-associated lncRNAs (CARs) were represented by at least one probe on the custom microarray, of which 64 showed significant changes in expression between normal and tumor samples (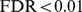). 43 CARs displayed consistent downregulation in breast cancer, while only 17 were found to be upregulated in tumor, and 4 CARs contained probes with inconsistent expression changes (Table S5). A total of 9 CARs overlapped with annotated lncRNAs, of which NEAT1, MALAT1, and MEG3 are known to be regulated by cancer pathways [19], [42], [54], [55]. Further, 9 CARs were found in introns of protein-coding genes (e.g. in introns of ARID5B, CALD1, EXT1), and 39 spanned introns and exons of a protein-coding gene (e.g. FOS, HNRNPH1). Expression levels of CARs appeared to be constant among Basal-like versus Luminal A and B tumor samples (Figure 3B and Table S4). Hence, CARs might be regulated by pathways being responsible for the onset and progression of primary tumors; however, not by pathways controlling the genesis of a particular subtype. The expression changes detected by the custom microarray of three randomly selected CARs were successfully validated by RT-qPCR (Figure S5).

### Differentially expressed non-coding transcripts showed low sequence conservation, but partly secondary structure conservation

Previous studies have indicated that the primary sequence conservation of lncRNAs is in general lower than for protein-coding genes [22], [23], [56]; however, a substantial fraction displays evidence for purifying selection on RNA structures in mammals [57]. Hence, we investigated, if non-coding DE-probes regulated in breast cancer are conserved on sequence and/or on secondary structure level.

Non-coding DE-probes showed less conserved sequence composition than DE-probes that mapped to protein-coding genes; however, they were more conserved than neutrally evolving sequences (Figure S6A). DE-probes significantly differentially expressed in the comparison of Basal-like versus Luminal A and B tumor samples displayed less sequence conservation than DE-probes significantly differentially expressed between normal and tumor tissue samples.

Assessing the structural conservation of non-coding DE-probes, we found these enriched for genomic loci with conserved structure motifs as predicted by SISSIz [53]; however, not for motifs predicted by RNAz [58] and Evofold [59] (Figures 2B, 3B, and Table S4). The three computational methods require different degrees of sequence conservation in the alignment, which may explain the observed imbalance. SISSIz predicts secondary structure motifs at loci with primarily low sequence conservation, while RNAz and Evofold reach optimal classification rates only for alignments with moderate or high sequence conservation, respectively [57], [60]. This result suggests an involvement of ncRNAs with structural motifs in breast carcinogenesis; however, only for those molecules with comparable low sequence conservation. Further, our studies revealed that DE-probes were in general less conserved in their sequence than probes with detectable secondary structure motifs (Figure S6B). Hence, many DE-probes corresponded to genomic loci for which reliable classification, whether the locus contains a secondary structure motif or not, is not possible due to low sequence conservation. Also, the detection of structured differentially expressed ncRNAs by microarrays is hampered by a lower affinity of those to array probes. This was reflected by lower signal intensities of probes, located in genomic loci with conserved secondary structure motifs compared to the remaining probes (Figure S6C).

### Differentially expressed non-coding transcripts were enriched in regulatory sites

The genomic location and frequency of non-coding DE-probes was assessed investigating promoters, enhancer regions, transcription factor binding sites, or transcriptionally active or in-active regions (see Table S7 for detailed information about annotations). Non-coding DE-probes, regulated between normal and tumor tissue samples, were enriched in promoter sites as defined by CpG islands and/or H3K4 trimethylation sites, in chromatin marks that are characteristic for enhancers (H3K4 monomethylation and H3K27 acetylation), and in genomic regions actively transcribed (Pol II binding sites, H3K36 trimethylation sites) (Figure 2D, Table S4).

The discrepancy between high (low) odds ratios for DE-probes upregulated (downregulated) in tumor samples and transcriptionally active sites (H3K36 trimethylated) - as opposed to DE-probes downregulated (upregulated) in tumor samples and transcriptionally inactive sites (H3K27 trimethylated) - might be a consequence of the unequal composition of ENCODE ChIP-Seq data, which is mainly derived from normal cell lines (Figure 2D, Table S4).

A less pronounced, but significant, enrichment was observed for transcription factor binding sites [7]. DNase-I hypersensitivity sites, characterized by regions where the chromatin is open in such a way that transcription factor binding is in general possible, were significantly enriched (Figure 2D).

Within tumor samples, non-coding DE-probes, which were upregulated in Basal-like tumors compared to Luminal A and B tumor samples, were significantly enriched in promoters (H3K4 monomethylation), enhancers (H3K27 acetylation), and in regions known to be actively transcribed (Pol II binding sites, H3K36 trimethylation, Figure 3D). Non-coding DE-probes downregulated in Basal-like tumor samples were significantly enriched in promoters (CpG islands and H3K4 trimethylation), enhancers (H3K27 acetylation), transcription factor binding sites, Pol II binding sites, and in DNase-I hypersensitivity sites.

In summary, we observed enrichment of differentially expressed lncRNAs in regulatory and epigenetically modified sites, which might interfere with expression of adjacent protein coding RNAs.

### Differentially expressed non-coding transcripts were located in close proximity to protein-coding genes

*Cabili et al. 2011* has suggested that intergenic lncRNAs are preferentially located in proximal regions of protein-coding genes [23]. To validate this result for lncRNAs regulated in cancer, we assessed the distance of all non-coding intergenic probes to their nearest protein-coding gene, independent of reading strand. In a comparative analysis of normal versus tumor tissue samples we found more intergenic non-coding DE-probes in close genomic proximity to protein-coding genes than expected from randomly selected intergenic regions preserving probe length of 60 bp (Figure S7A, Kolmogorov-Smirnov test 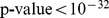).

For intergenic non-coding DE-probes significantly differentially expressed between Basal-like versus Luminal A and B tumor samples a similar distance distribution to neighboured transcription start sites of protein-coding genes was observed (Kolmogorov-Smirnov test 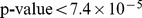); however, with a larger shift to proximal regions of protein-coding genes for DE-probes upregulated in Basal-like tumor samples (Figure S7B).

### Differentially expressed non-coding transcripts and nearest protein-coding genes displayed non-synonymous expression changes

Our results of significantly regulated non-coding loci (abundantly located in proximal regions of protein-coding genes, and enriched in regulatory sites) together with reports of previous studies about frequent *cis*-regulatory mechanisms of lncRNAs [14], [26], [27], [61], [62] encouraged us to analyze the expression variation for all non-coding DE-transcripts in comparison to their nearest protein-coding gene (Gencode v12). Exclusion criteria for potentially non-annotated distant exons of protein coding genes were: (1) If the non-coding DE-probe and the protein-coding gene were located on the same reading strand, only those lncRNA-mRNA pairs were accepted that exhibited significant expression changes in opposite directions. (2) If the non-coding DE-probe and the protein-coding gene were located on different reading strands, all lncRNA-mRNA pairs with significant expression changes were accepted.

**Figure 4:**
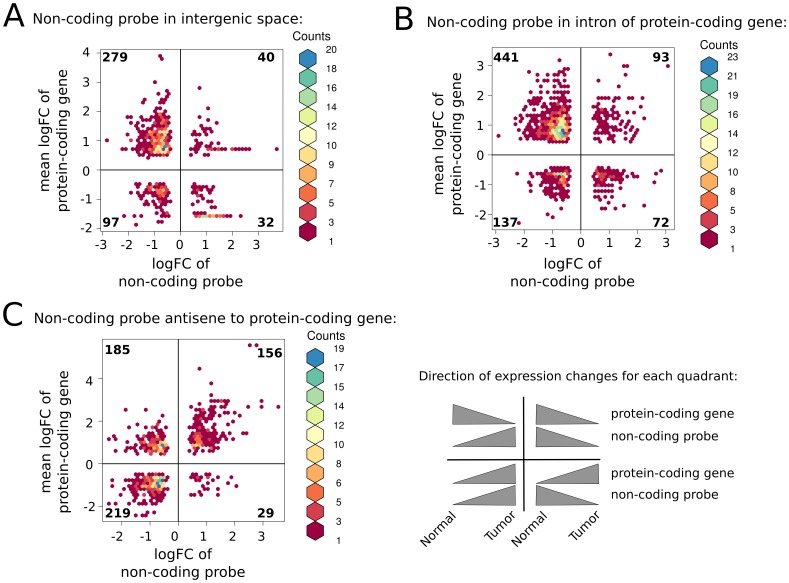
Proximal lncRNA – mRNA pairs. For non-coding DE- probes significantly differentially expressed between normal and tumor samples (FDR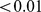) the protein-coding gene (Gencode release v12) with closest genome coordinates was identified, and the pair retained if the protein-coding gene was differentially expressed at the same FDR cutoff. Log2 fold change of the non-coding probe (*x*-axis) and the maximal log2 fold change of probes located in exons of the protein-coding gene (*y*-axis) is depicted as a bivariate histogram using hexagonal binning (R package hexbin). Pairs with converse fold changes are shown in the left upper and right lower quadrant. Pairs with consistent fold changes but opposite reading direction are shown in the left lower and right upper quadrant (see also panel describing direction of expression changes for each quadrant). Numbers in quadrant correspond to number of unique genes depicted. (**A.**) Proximal pairs, where the non-coding probe is intergenic. (**B.**) Pairs where the non-coding probes is in an intron of the protein-coding gene. (**C.**) Pairs where the non-coding probe and the protein-coding gene are on opposite strands and overlap at least partially.

We detected 416 protein-coding genes in nearest genomic proximity of 782 intergenic non-coding DE-probes, where both displayed significant differential expression variation between normal and breast tumor tissue (intergenic lncRNA-mRNA pairs, 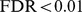, Figure 4A). The majority (75%) of those protein-coding genes showed non-synonymous expression changes with at least one intergenic non-coding DE-probe: 279 (32) protein-coding genes were found upregulated (downregulated) in tumor in relation to their nearby downregulated (upregulated) intergenic non-coding DE-probes in contrast to 137 protein-coding genes with synonymous expression variations (Figure 4A).

Further, a total of 1276 actively transcribed and putatively regulatory non-coding transcripts in introns of 655 regulated protein-coding genes were identified (intronic lncRNA-mRNA pairs, Figure 4B). Again, the majority of those protein-coding genes displayed non-synonymous expression changes with at least one intronic non-coding DE-probe. We found 441 (72) protein-coding genes upregulated (downregulated) in tumor while at least one intronic DE-probe was downregulated (upregulated).

**Figure 5:**
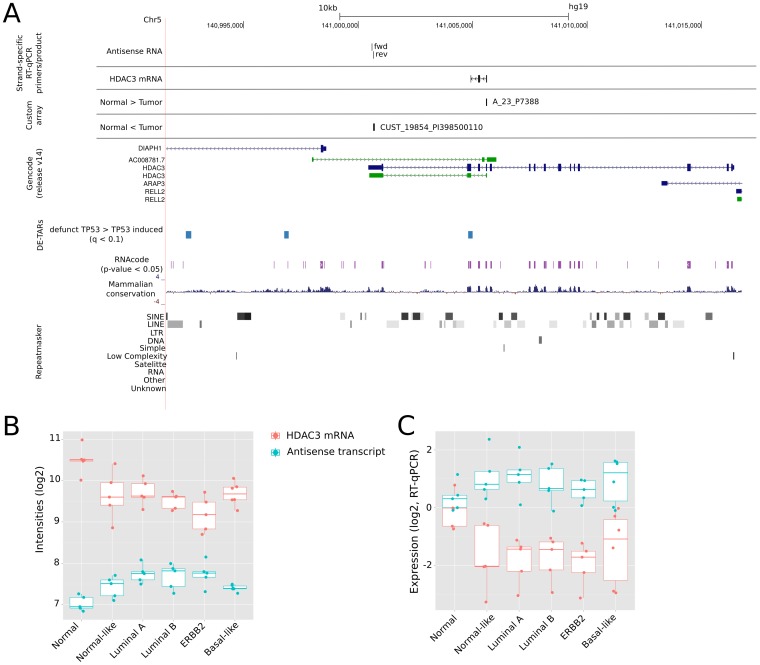
HDAC3 (histone deacetylase 3) mRNA and its putative regulatory antisense lncRNA. (**A.**) Genomic locus of HDAC3 on chromosome 5 and the antisense transcript downstream of HDAC3 with genomic positions of strand-specific RT-qPCR primers/products. Annotation track DE-TAR corresponds to genomic loci significantly downregulated upon TP53 induction [43]. Both transcripts appear to be significantly differentially expressed on the custom microarray (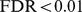), exhibiting a non-synonymous expression pattern (**B.**). The transcription start site of the annotated antisense RNA overlaps with the transcription start site of DIAPH1. Genome-wide predictions of functional open reading frames (RNAcode, 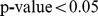) correspond mainly to exons of HDAC3 mRNA, while some short putative open reading frames overlap the antisense transcript. (**C.**) Strand-specific RT-qPCR validations relative to normal sample “RP38” for both, the HDAC3 mRNA and the antisense transcript.

For non-coding DE-probes antisense to protein-coding genes, we observed a total of 865 lncRNA-mRNA pairs comprising 565 unique protein-coding genes (Figure 4C). Here, we identified a balanced fraction of pairs with synonymous or non-synonymous expression changes with the exception of a small number of protein-coding genes (29) that were upregulated in tumor and with an antisense lncRNA downregulated. An example of a protein-coding gene downregulated in breast cancer with a non-coding antisense transcript significantly upregulated in breast cancer versus normal tissue is HDAC3 (histone deacetylase 3) on chr5 (Figure 5). HDAC3 belongs to the class I of histone deacetylase family of proteins, which regulates gene expression by histone deacetylation at promoter sites thus transforming the chromatin into a more “packed” state (heterochromatin) leading to transcriptional repression of genes.

A KEGG pathway enrichment analysis for all protein-coding genes, which were significantly differentially expressed between normal and tumor tissue and which were a member of the above described lncRNA-mRNA pairs revealed many different cancer-related pathways among the significant KEGG terms (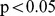, Table S6).

We observed a less pronounced number of lncRNA-mRNA pairs for the comparison of Basal-like versus the Luminal A and B tumor samples (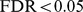). Only 15 protein-coding genes with nearby non-coding DE-probes in intergenic space were detected, 17 with non-coding DE-probes in their introns, and 117 with non-coding DE-probes in antisense direction.

In summary, these findings directly support the hypothesis that expression of protein-coding genes associated to cancer relevant pathways might be affected by lncRNAs located in close genomic proximity.

## Discussion

### The landscape of non-coding transcription shows dramatic changes in breast cancer

Breast cancer is a highly heterogeneous disease with distinct molecular subtypes characterized by patterns of protein-coding gene expression [2], [3] and genomic DNA alterations [50], [63]. The genomic changes may contribute to alterations at the expression levels; tumors of the same mRNA expression subtype often share similar copy number variations, with the most prevalent genomic alterations observed for the Basal-like and ERBB2 subtypes [63]–[65]. Further, it has been demonstrated that 12% to 40% of the expression variation of the protein-coding genes may be explained by genomic alterations [50],[66]. We investigated whether the observed differential expression of lncRNAs is merely a consequence of genomic aberrations. We found that the observed correlation between copy number variations and lncRNA expression was very low, only 6% of the observed expression changes may be explained by copy number variation. Thus, and in opposite to previous findings for protein-coding genes [49], [50], we conclude that the majority of the observed differential expression of lncRNAs is influenced by other factors than underlaying DNA aberrations.

Overall, 9647 unique non-coding loci were significantly differentially expressed between 26 breast tumor and 5 normal tissue samples. These non-coding transcripts were located in the intergenic space (3194) and in introns of protein-coding genes (5088), further in antisense or bidirectional genomic location relative to protein-coding genes (1365). Several of the detected differentially expressed lncRNAs were well described members of cancer-related pathways, including tumor suppressors and oncogenes (e.g. MEG3 [55], Xist [67], [68], MALAT1 [19], [42], H19 [39], [69], GAS5 [40], and HOTAIR [25], [70]). However, the majority of differentially expressed lncRNAs corresponded to novel transcripts of unknown function. Differentially expressed mRNAs were enriched in cancer- but not in adipocyte-specific KEGG pathways, suggesting that the observed differences between normal and tumor tissue samples are not histological artefacts. We therefore conclude that the observed differential expression of lncRNAs is mainly reflecting the difference between normal and tumorous epithelial cells and is thus associated with breast cancer.

LncRNA differential expression between tumor subtypes was on a much lower scale than between normal and tumor tissue. This may be a consequence of smaller effect sizes and larger within group heterogeneity combined with comparably smaller sample sizes. However, it might also reflect a bias of the custom array. Apart from database derived lncRNAs it contained in house identified lncRNAs from cell cycle, oncogenic, and tumor suppressor pathways. Alterations in these processes are common to many tumors, but transcripts associated with breast cancer subtypes, like estrogen- or progesterone receptor controlled lncRNAs, are likely underrepresented on the array.

Investigating evolutionary conservation of tumor-related non-coding RNAs we observed that selective pressure on primary sequence is small compared to protein-coding exons, which is in line with reports by others [22], [23], [56]. In addition, we detected conservation of secondary structure motifs in mammals for about 10% of non-coding DE-regions regulated between normal and breast tumor tissue (9.8%) and between Basal-like versus Luminal A and B tumor samples (11.5%). This fraction was only marginally explained by the observed number of regulated short ncRNAs usually folding into distinct secondary structures. Given that the majority of DE-probes were less conserved in primary sequence than it is required for optimal signal-to-noise ratios in RNA secondary structure motif detection tasks, a notable number of conserved structure motifs may still be hidden among our set of novel non-coding tumor-related non-coding RNAs. Further, we noticed on average less sequence conservation for lncRNAs differentially expressed within tumor tissue (Basal-like versus Luminal A and B tumor samples) than for lncRNAs differentially expressed between normal and tumor tissue.

### Non-coding RNAs may regulate transcription of protein-coding genes in *cis*

Several lncRNAs have been found to regulate gene expression in *cis* [14], [26], [27], [61] or in enhancer regions [71]. We hypothesize that *cis* acting lncRNAs regulate expression of tumor suppressors and (to a lesser extent) oncogenes in a direct or indirect way, and thus contribute to tumorigenesis. Overall, we found a significantly smaller distance of differentially expressed non-coding regions to the proximal protein coding gene than expected by randomly drawing from the transcripts represented on the custom array. We detected a massive deregulation of non-coding transcription from regulatory DNA sites upstream of protein coding sequences. These may partly correspond to short RNAs (50bp to 200 bp length) that are transcribed upstream of coding genes, which are known to be a target of polycomb proteins and can induce repression of coding genes in *cis* [72].

Further, we identified 311 protein-coding genes with proximal long non-coding RNAs in intergenic space and non-synonymous expression changes, suggesting that these lncRNAs may interfere with mRNA expression. Synonymous pairs were only assessed for RNAs on opposite strands to avoid counting of unknown mRNA exons and these synonymous pairs were significantly fewer than non-synonymous pairs. For the majority of non-synonymous pairs the protein-coding gene was found down- and the intergenic lncRNA was identified being upregulated in tumors samples. We noticed an enrichment of tumor suppressor protein-coding genes for mRNAs downregulated in tumor. Together with the fact that the majority of mRNAs were down- and the majority of non-coding transcripts were upregulated in tumors one may speculate whether the upregulation of lncRNAs contributes to the downregulation of tumor suppressor genes and thus to the progress of cancer.

In few cases, non-synonymous expression in opposite reading direction, exhibiting overexpression of breast cancer-associated mRNAs along with an antisense lncRNA downregulated in tumor samples, was detected. *Cabili et al. 2011* found that the expression patterns of lincRNAs and their nearest protein-coding neighbour are not more correlated than pairs of two neighbouring protein-coding genes [23]. Thus, synonymous pairs may just reflect that transcription of a particular genomic locus is accompanied by transcription of neighboured loci because the surrounding chromatin is open [73]. However, lncRNAs can positively affect the transcription of adjacent genes by inducing chromatin looping between the ncRNA loci and the neighbouring genes [74]. *Ørom et al. 2010* report intergenic non-coding RNAs acting as enhancers for their nearest protein-coding gene, which results in synonymous expression changes between non-coding RNAs and mRNAs [14]. In summary, our results suggest that lncRNA s may act in the regulatory control of adherent cancer-related genes and their malfunction may present an important factor in the development and progression of breast cancer.

An example for a strongly anticorrelated, non-synonymous pair with a tumor-downregulated mRNA is HDAC3 and its antisense lncRNA transcript. We found HDAC3 expression in normal samples and strongly downregulated but detectable in tumor tissue samples. Class I histone deacetylases, like HDAC3, are repressing the transcription machinery for various genes in cancer [75]. On the other hand, studies reported that HDAC3 is an inhibitor of migration of metastatic breast cancer cells [76].

### Chromatin-associated lncRNAs may contribute to epigenetic changes during carcinogenesis

In the breast cancer samples used for our investigations, we observed a massive downregulation of lncRNAs previously described to be a component of chromatin [27]. Chromatin-associated lncRNAs (CARs) have been found to regulate transcription in *cis* by mediating chromatin modifications in close genomic proximity. We identified downregulated CARs in vicinity or overlapping the oncogenes IGF1R, MYLK [77], the breast cancer drug target OGT (O-GlcNAc transferase) [78], and the breast cancer-related cyclin CCNL1 [79]. CARs also overlap with lincRNAs that were found to bind to PRC2 (polycomb repressive complex 2) or RCOR1 (REST corepressor 1) and regulate transcription in *trans* through establishing repressive chromatin marks at distant sites [27]. We found 5 of these CARs/lincRNAs (MALAT1, MEG3, NEAT1, NBPF1, and AC058791.1) significantly downregulated in primary tumor samples compared to normal tissue. The observed downregulation of CARs in tumor may thus be responsible for a reprogrammed chromatin state in breast cancer leading to the activation of oncogenes or repression of tumor suppressors in *cis* or *trans*.

In summary, the observed massive deregulation of lncRNAs may be an important characteristic of breast cancer development and progression. The frequently observed anticorrelation between lncRNAs and adjacent breast cancer relevant onco- and tumor suppressor genes may give rise to novel drug targets at the non-coding RNA level. Finally, the perturbation of chromatin-associated lncRNAs encourages for a more detailed investigation of the role of lncRNAs in epigenetic reprogramming in breast cancer etiopathology.

## Materials and Methods

### Ethics Statement

All studies are approved by the Norwegian Regional Committee (REC) for Medical and Health Research Ethics (REC South East, reference numbers S97103 and 429-04148). All patients are informed and have declared written informed consent that their samples are used for research.

### Tissue Samples

Fresh frozen tumor biopsies from early breast cancer cases were collected from 920 patients included in the Oslo Micrometastasis (MicMa) Study - Oslo I from various hospitals (a collaboration between Buskerud-, Bærum-, and different sections at the Oslo University Hospital, Norway) between 1995 and 1998 [28], [44], [80]. A total of 26 breast carcinomas have been selected for lncRNA expression study using the nONCOchip microarray. The samples have been classified into five clinically relevant tumor subclasses based on their mRNA expression levels [44], [81]. In addition, 5 breast tissue samples from breast reduction operations were provided from the Colosseum Clinic, Oslo in co-operation with Akershus University Hospital, Lørenskog and are herein defined as being normal breast tissue.

### Custom expression microarray design - nONCOchip

We used the nONCOchip, a custom-designed Agilent microarray containing 203,527 probes covering protein-coding and non-coding genomic loci (GEO accession number GPL13648). The nONCOchip interrogates probes for lncRNAs regulated by three tumor-relevant pathways (mitotic cell cycle, anti-proliferative and pro-apoptotic p53, and pro-proliferative and anti-apoptotic Stat3), known lncRNAs, as well as mRNAs [43].

In detail, probes of 60bp length have been designed following Agilent's standard design protocol for expression exon microarrays as available from eArray (https://earray.chem.agilent.com/earray/). eArray design was performed according to the base composition methodology where probes are equally distributed across the target sequence, and uniqueness of probes have been checked against all known RefSeq (m)RNAs. Target sequences were grouped into three length categories defining the required number of probes. Target sequences of length 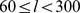 are represented by exactly one probe, while target sequences of length 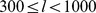 are represented by up to five probes. Target sequences longer than 1000bp were split into intervals of 1000bp and number of probes selected according to length of subsequence. For target sequences of unknown reading strand, i.e. sequences originating from the transcriptome-wide study of cancer-related pathways [43] or from ncRNA predictions [57], [59], probes for the plus and minus strand have been designed. It should be noted that probes of 60bp length exclude the detection of the majority of smaller ncRNAs, like mature miRNAs.

### Custom expression microarray processing

All samples assembled for expression analysis with the nONCOchip custom microarray were prepared for microarray performance using the Agilent Quick Amp Labeling Kit for single color following manufacturer's instructions. RNA quality was checked using Agilent's 2100 Bioanalyzer; only samples with a 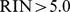 entered the processing. Instead of the Oligo-dT/T7-primer delivered with the Quick Amp Labeling Kit, we used 120 pmol of a random N6/T7 primer synthesized by Metabion. 1* µ*g RNA was used as input for the labeling procedure. cRNA quantity was checked using a NanoDrop ND-1000 UV-VIS Spectrophotometer, as enlisted in the manufacturers instructions. 1.5* µ*g of labeled cRNA was used for hybridization with the 244k custom microarray following manufacturers instructions. After hybridization the arrays were washed according to the manual and scanned using the Axon GenePix 4200 Scanner and GenePix Pro 6.1 Scan software with the following settings for scanning: 100% laser power; focus 0; 5* µ*m pixel size; 2 lines to average; wavelength at 532 nm with standard green filter. Result tables were extracted after grid placement using GenePix Pro 6.1 Software. Result tables were used for subsequent data analysis.

### Custom expression microarray data analysis

Differentially expressed probes were identified by using R [82] and the Bioconductor library limma [83]. Quality control of arrays were performed by checking distribution of “bright corner”, “dark corner” probes, and relative spike-in concentration versus normalized signal. The controls confirmed high quality of the results and consequently all microarray data were included in the downstream analysis. To retrieve a set of probes mapping to unique genomic positions in hg19 we used BLAT [84] with the parameter -minIdentity = 93 allowing to detect probes spanning splice sites. All probes mapping to more than one distinct genomic region were discarded. Normalization between arrays was done by using quantile normalization [85]. In order to reduce the number of *t*-tests nonspecific filtering was applied as follows: The expression of a probe must be larger than background expression in four arrays. Background expression is defined by the mean intensity plus three times the standard deviation of negative control spots (Agilent's 3xSLv spots). In addition, a probe must exhibit a nonspecific change of expression of at least IQR 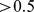. Finally, a linear model was fitted using the R package limma and reliable variance estimates were obtained by Empirical Bayes moderated t-statistics. False discovery rate was controlled by Benjamini-Hochberg adjustment [86].

### Quality control of the custom expression microarray

For quality control of the expression data, we investigated whether the subtype classification obtained in previous studies for the same set of tumor samples was reproducible using the nONCOchip. Gene expression of PAM50 genes was compared to previous profiling of the same samples using Agilent's Whole Human Genome Oligo 44k Microarrays [81]. High concordance to the original subtype assignments for all tumor samples was revealed depicting sufficient Pearson's correlation coefficients (Figure S1A). The results indicate high molecular reproducibility for mRNA expression profiles applying the nONCOchip, thereby suggesting high qualitative performance for lncRNA expression profiling.

### aCGH microarray data analysis

The genotypes aCGH dataset has been published previously [87]. For each sample, the copy number data were log2-transformed, segmented, and probe values were replaced by segment averages, using the Piecewise Constant Fitting (PCF) algorithm [63], [88]. In order to obtain matching copy number and expression data sets, the following procedure was applied to each sample and to each of the non-coding transcripts in the dataset to obtain a corresponding copy number value: A PCF value was found for each of the expression probe position through interpolation of the piecewise constant regression function. The average PCF value over all expression probes associated with a particular probe is calculated. This average then defines the copy number value for that probe in the given sample.

### Defining a set of non-coding probes

A *bona fide* set of non-coding probes in intergenic and intronic regions was constructed from the significantly differentially expressed probes as follows: (1) All probes overlapping with at least one nucleotide with protein-coding exons (independent of reading strand) as annotated in Gencode release v12 [89], UCSC genes [90], RefSeq [47], or Ensembl genes [91] were discarded. (2) Probes overlapping with a significant RNAcode [46] segment (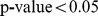) contain de-novo short open reading frames and were discarded from the set of non-coding probes. In detail, genome-wide Multiz alignments [92] for 46 vertebrate genomes have been downloaded from http://hgdownload.cse.ucsc.edu/goldenPath/hg19/multiz46way/ and split into shorter alignments of at most 400bp length to enable parallel processing. Each multiple alignment was assessed by RNAcode in all six possible reading frames, and the longest segment in the reference sequence of maximal score reported. All segments with an RNAcode 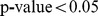 define de-novo protein-coding regions. We refrained from adjusting p-values for multiple testing, as we are not interested in a set of highly reliable protein-coding segments (i.e. reducing the number of false positives), but in reducing the number of regions falsely interpreted as non-coding (i.e. reducing the number false negatives). An RNAcode p-value of 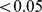 resulted in 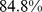 sensitivity (according to known protein-coding exons annotated in Gencode v12) and 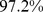 specificity (according to 10,000 sampled intergenic intervals preserving length distribution and repeat content of protein-coding exons). (3) All probes which have not been classified by RNAcode (due to low sequence conservation) but overlap with at least one nucleotide genomic regions of translated sequences patterns similar to amino-acid sequences of known human proteins may be of coding origin and, hence, were discarded (tblastn [93] with parameters -word-size 3 -evalue 0.05 and RefSeq database from March 7, 2012). Applying these filters results in 53,577 *bona fide* non-coding probes in intergenic regions and 71,228 in introns of protein-coding genes. For simplicity, we termed probes passing these three filters as non-coding probes.

Probes antisense to a protein-coding exon, but not containing a significant RNAcode hit (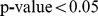) on the same strand define a separate set of antisense non-coding probes comprising in total 14,272 probes.

### Statistical analysis of annotation overlaps

Overlap with annotation sets was calculated using R [82] and the Bioconductor library genomeIntervals [94]. We further used the R library Snow to enable parallel processing [95]. For each contrast of interest the overlap with a particular annotation set was computed in terms of (1) absolute number of differentially expressed probes (DE-probes) overlapping with a particular annotation and (2) the odds ratio of observed relative overlap versus relative overlap of a background list consisting of all probes on the nONCOchip. Observed odds, randomized odds and odds ratios are defined as follows:
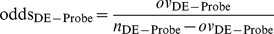
(1)

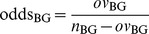
(2)

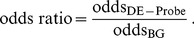
(3)


The observed number of overlapping probes is given as 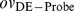, while 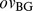 corresponds to the number of overlapping probes in the background list. The overall number of DE-probes is denoted as 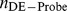, and the overall number in the background list as 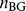. Significance of odds ratios was assessed by using Fisher's exact test as implemented in R. We also report the 95% confidence interval of the odds ratio, which is larger than 1 in case of an enriched number of overlapping nucleotides and less than 1 in case of depletion. A probe was interpreted as an overlapping probe if it maps to at least 90% to an interval of the according annotation set.

### Annotation categories

A detailed description and listing of annotations sets used is given in Methods S1 and in Table S7.

### KEGG pathway enrichment analysis

KEGG pathway enrichment analysis was performed using the R library GOstats [96] with pathway annotations as stored in *KEGG.db* and *org.Hs.eg.db*. Significance of enrichment was assessed by a one-sided hypergeometric test where the universe contains all genes of the nONCOchip which passed nonspecific filtering.

### Validation of lncRNA expression analysis by RT-qPCR

For quantitative real time PCR 250 ng of total RNA was reverse transcribed using random primers and the High-Capacity cDNA Reverse Transcription Kit following the manufacturer's recommendations (Applied Biosystems, now Life Technologies). Transcript expression analysis was performed using Fast SYBR Green Master Mix according to the manufacturer's instructions (Applied Biosystems, now Life Technologies). 5* µ*l of 1∶12.5 diluted cDNA was used per reaction. Experiments were performed in triplets and all transcript quantification data were normalized to *β*-Actin mRNA. All primers were designed using Primer3 (v0.4.0, default parameters, Table S8). The UCSC In-Silico PCR option (http://genome.ucsc.edu/cgi-bin/hgPcr) was used to check for unspecific primer matches.

## Supporting Information

Figure S1
**Expression changes recovered mRNA based subtype classification, and were independent of copy number changes.** (**A.**) Heatmap of Pearson's correlation coefficients of expression levels of the protein-coding genes that define the five known mRNA subclasses of breast cancer on the custom microrarray compared to expression levels of these genes in the same set of tissue samples on Agilent 4×44k arrays [28], [44]. Correlation was calculated for 44 of the 50 protein-coding genes defining the mRNA expression breast cancer subclassification. The remaining 6 genes were not represented on the microarray due to the requirement, that at least one probe per gene must be mapped to a unique genomic locus (hg19). PAM50 gene expression is highly correlated between same tumor samples on the two different expression arrays. Abbreviations LA, LB, Bl, and Nl indicate Luminal A, Luminal B, Basal-like, and Normal-like tumor subtypes, respectively. (**B.**) Smoothed scatterplot of expression changes and copy number variations for non-coding DE-probes regulated between normal and tumor samples (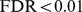). The portion of probes with similar expression changes and similar copy number variation is reflected by different blue shades. Black dots mark extreme values, and red lines correspond to average intensities for positive and negative expression changes. Spearman's correlation coefficients of 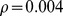 and 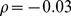, respectively, indicate that the contribution of the copy number changes have only marginal effect on the expression variation of non-coding regions.(PDF)

Figure S2
**Differential expression of non-coding probes in breast tumor.** Heatmap of non-coding probes with significant expression variation between molecular tumor subtypes (F-test with moderated residual mean squares – limma R library, 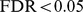). Clinical data indicate disseminated tumor cell status (**DTC**, 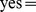
disseminated tumor cells detected, no = not detected); age at onset (**Age**); histological grade 1, 2 or 3 (**Grade**); TP53 mutational status (**TP53**, 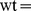
wild-type and 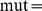
mutated); status of epidermal growth factor receptor 2 (**Her2**, 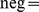
Her2 negative, 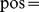
Her2 positive); status of progesterone receptor (**PR**, 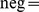
PR negative, 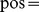
PR positive); and status of estrogene receptor (**ER**, 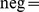
ER negative, 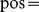
ER positive).(PDF)

Figure S3
**Unsupervised clustering of tumor samples.** Hierarchical clustering of probes passing unspecific filtering, i.e. 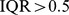 between tumor samples and expression above the background in at least four arrays. (**A.**) Hierarchical cluster tree of probes located in exons of protein-coding genes (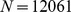), and (**B.**) of non-coding probes (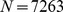). Variance within clusters was minimized by applying Ward's method on scaled 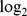 intensities of probes, and correlation was used as distance function. Uncertainty of clusters was assessed by bootstrapping with 10,000 iterations (R package pvclust). Red numbers indicate cluster reliability in percent, here 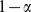 with 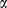 being the significance level to reject the null hypothesis that the cluster is not present in the data. Variation explained by array processing batches was removed prior to clustering (R package limma – removeBatchEffect) in order to receive a clustering of samples which is solely based on biological variation. Detailed description of clinical, pathological and immunohistochemical data of presented tumor samples is provided in caption of Table S1.(PDF)

Figure S4
**Differential expression of lncRNAs.** Heatmap of lncRNA (Gencode v12) expression changes between normal and tumor tissue. For each lncRNA and patient sample, the median expression of all significantly differentially expressed probes (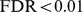) located in exons of the lncRNA is depicted. Clinical data indicate disseminated tumor cell status (**DTC**, 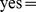
disseminated tumor cells detected, 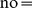
not detected); age at onset (**Age**); histological grade 1, 2 or 3 (**Grade**); TP53 mutational status (**TP53**, 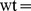
wild-type and 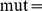
mutated); status of epidermal growth factor receptor 2 (**Her2**, 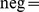
Her2 negative, 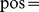
Her2 positive); status of progesterone receptor (**PR**, 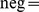
PR negative, 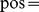
PR positive); and status of estrogene receptor (**ER**, 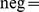
ER negative, 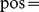
ER positive).(PDF)

Figure S5
**RT-qPCR validation of differentially expressed chromatin-associated lncRNAs.** Subsequent analysis of three chromatin-associated lncRNAs (CARs, Table S5) [27] chosen for validation. Validation was performed using all original RNA samples by RT-qPCR. Plots for the chromatin-associated lncRNAs CAR-CALD1 (spanning intron of CALD1 mRNA), CAR-HNRNPH1 (spanning introns and exons of HNRNPH1 mRNA) and CAR-FTX (spanning introns and exons of lincRNA FTX) depict changes in expression (log2 scale). Sample types are represented by different colours: normal breast tissue (yellow); Luminal A subtype (dark blue); normal-like samples (green); the basal-like subtype (red); the ERBB2 samples (purple) and the Luminal B subtype (light blue). The 2D matrix represents the p-value after testing for the different hypotheses (p-value
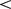
0.01 = **; p-value
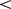
0.05 = *).(PDF)

Figure S6
**Sequence conservation and hybridisation intensities.** Empirical cumulative distributions (ECDF) of average PhastCons scores of DE-probes (Normal vs. Tumor with 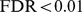, Basal-like vs. Luminal tumors with 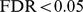) either compared to neutral evolving sequences preserving length distribution of coding exons (**A.**) or to array probes located in genomic loci with conserved secondary structures – RNAz [57], [58], SISSIz [53], [57], and Evofold [59] (**B.**). (**C.**) ECDF of maximal microarray hybridisation intensities of probes located in loci with conserved secondary structure motifs compared to all remaining probes on the custom microarray.(PDF)

Figure S7
**Genomic distance of intergenic non-coding DE-probes to protein-coding genes.** Empirical cumulative distribution function (ECDF) of genomic distances of intergenic non-coding probes either significantly differentially expressed between tumor and normal samples (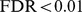, **A.**) or between Basal-like and Luminal tumors (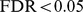, **B.**) to their nearest protein-coding gene (Gencode v12), not taking the reading direction into account.(PDF)

Table S1
**Clinical, pathological, and immunohistochemical data of presented breast tumor samples.** Column headings indicate sample identifier (**Sample ID**); Age at onset (**Age**, years with one decimal); tumor cell content in percentage (**TCC**, 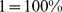, n/a if not available); tumor size in cm (**Tumor Size**); status of breast tumor (**Tumor Status**, 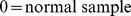, 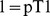 [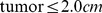], 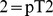 [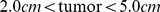], 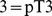 [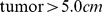], 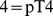 [infiltrating skin or thoracic wall], 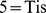 [Carcinoma in situ], 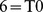 [no detected primary tumor], 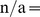
not available); histology (**Histology**, 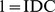 [invasive ductal carcinoma], 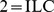 [invasive lobular carcinoma], 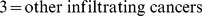, 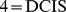 [ductal carcinoma in situ]); histological grade 1, 2 or 3 (**Grade**); status of estrogene receptor (**ER status**, 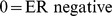, 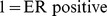); status of progesterone (**PR status**, 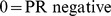, 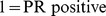); HER2 status – combination of IHC and FISH (**HER2 combined**, 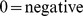 [either 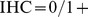 or 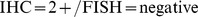], 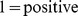 [either 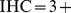 or 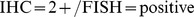], n/a
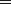
missing); TP53 mutational status (**TP53 status**, 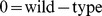, 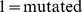); disseminated tumor cell status (**DTC status**, 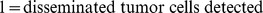, 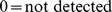); PAM50-based tumor subtype (**PAM50 subtype**, 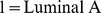, 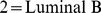, 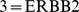, 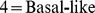, 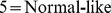) and 44k mRNA expression-based subtype (**Tumor subtype**, 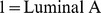, 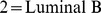, 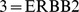, 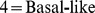, 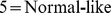). Details of DTC detection are further described in *Wiedswang et al. 2003* [80].(PDF)

Table S2
**KEGG pathway enrichment analysis for protein-coding genes significantly differentially expressed (Normal versus Tumor).** Most enriched KEGG pathways (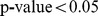) of genes significantly differentially expressed between normal and tumor samples (Gencode release v12, 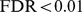). Column headings indicate ID of KEGG pathway (**ID**), significance of enrichment (**P-value**), odds ratios (**Odds ratio**), expected number of genes associated with tested pathway (**Exp. count**), number of significantly differentially expressed genes associated with this pathway (**Count**), number of genes from the gene universe that are annotated in that pathway (**Size**), name of the pathway (**Pathway Name**), and a list of genes which are regulated in that pathway and were significantly differentially expressed. Analysis was done by using the Bioconductor GOstats package. Mapping of genes to Entrez IDs is based on the NCBI gene information table (version: July 1, 2012). Significance of enrichment was assessed by a one-sided hypergeometric test where the universe contains all genes of the custom microarray which passed unspecific filtering (Materials and Methods).(PDF)

Table S3
**Known non-coding RNAs differentially expressed between normal versus tumor samples.** Summary of known non-coding RNAs (Gencode v12) significantly differentially expressed between normal and tumor patient samples (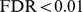). Column headings indicate official Gencode gene name (**Gene name**), Gencode identifier (**Gene ID**), if ncRNA transcript is *bona fide* non-coding (***Bona fide*** **non-coding**, for detailed description of filter refer to Methods S1), Gencode transcript ID (**Transcript ID**), position of transcript in human genome version hg19 (**Position of transcript**), position of probe in human genome version hg19 (**Position of probe**), custom array probe ID (**Probe ID**), and fold change in log2 scale (**logFC**). A fold change of 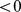 indicates 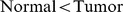, and a fold change of 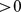 denotes 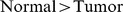.(XLSX)

Table S4
**DE-Probes overlap with genomic annotation.** Number of DE-Probes significantly differentially expressed between normal and tumor samples (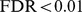) or Basal-like and Luminal tumors (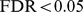), and overlapping with diverse sets of genomic annotation. Annotation datasets are described in Table S7. Overlaps are calculated by using the Bioconductor genomeIntervals package [94]. The significance of the observed overlap is assessed by calculating odds ratios of observed (DE-Probes) versus expected (all probes on microarray) relative overlaps. Odds ratios are calculated and tested by Fisher's exact test for significant enrichment or depletion (see Materials and Methods). Column heading **Annotation** indicates annotation datasets for which overlap is computed, and **Survey** corresponds to the direction of expression variation (either 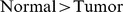: overlap for probes downregulated in tumor, 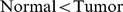: overlap for probes upregulated in tumor; or 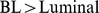: overlap for probes upregulated in Basal-like tumors, 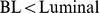: overlap for probes downregulated in Basal-like tumors). Remaining columns indicate the results (**Odds ratio**, **P-value**, and 95% confidence interval for odds ratio − **95%CI**) and the data (**DE-Probes**: number of differentially expressed probes located in annotation, i.e. fraction of overlapping probe nucleotides 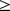 0.9, or non-overlapping with annotation, i.e. fraction of overlapping probe nucleotides 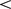 0.9; **BG**: number of array probes located in annotation, i.e. fraction of overlapping probe nucleotides 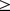 0.9, or non-overlapping with annotation, i.e. fraction of overlapping probe nucleotides 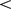 0.9) of Fisher's exact test.(PDF)

Table S5
**Chromatin-associated lncRNAs differentially expressed between normal versus tumor.** Summary of chromatin-associated lncRNAs [27] significantly differentially expressed between normal and tumor patient samples (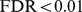). Column headings indicate if chromatin-associated lncRNA (CAR) is *bona fide* non-coding (***Bona fide*** **non-coding**, for detailed description of filter refer to Methods S1), position of CAR in human genome version hg19 (**CAR - Genomic locus**), genes the CAR overlaps with (**CAR - overlaps gene**, in detail Gene name;gene type;reading strand), position of probe in human genome version hg19 which overlap CAR in either reading direction (**Probe - Genomic locus**), custom array probe ID (**Probe - ID**), fold change in log2 scale (**Probe - logFC**), and genes the custom array probes overlap with (**Probe - overlaps gene**). A fold change of 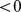 indicates 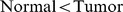, and a fold change of 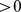 denotes 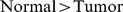. Since reading strand of CARs is unknown we report all probes overlapping with a CAR no matter of reading direction. Abbreviations used for gene and transcript types are as follows: **AS** (antisense), **NC** (lincRNA, miRNA, snRNA, snoRNA, scRNA, non coding, miscRNA), **NMD** (nonsense mediated decay), **PC** (protein coding), **PG** (pseudogene), **PT** (processed transcript), **RI** (retained intron), **SI** (sense intronic), and **SO** (sense overlapping).(XLSX)

Table S6
**KEGG pathway enrichment analysis for mRNAs with intergenic, intronic, or antisense non-coding DE-probes.** Most enriched KEGG pathways (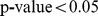) of significantly differentially expressed protein-coding genes (Gencode release v12, 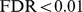) with a non-coding DE-probe (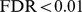) either located in intergenic space and proximal to the protein-coding gene, located in intron of the protein-coding gene, or antisense to the protein-coding gene. Column headings indicate ID of KEGG pathway (**ID**), significance of enrichment (**P-value**), odds ratios (**Odds ratio**), expected number of genes associated with tested pathway (**Exp. count**), number of significantly differentially expressed genes associated with this pathway (**Count**), number of genes from the gene universe that are annotated in that pathway (**Size**), name of the pathway (**Pathway Name**), and a list of genes which are regulated in that pathway and significantly differentially expressed. Analysis was done by using the Bioconductor GOstats package. Mapping of genes to Entrez IDs is based on the NCBI gene information table (version: July 1, 2012). Significance of enrichment was assessed by a one-sided hypergeometric test where the universe contains all genes of the custom microarray which passed unspecific filtering (see Materials and Methods).(PDF)

Table S7 **Detailed documentation of used annotation categories.** Column headings **Annotation**, **Abbreviation**, **Source/URL**, **Assembly**, **Citation**, and **Comment** indicate the according genomic feature, the abbreviation used in figures and tables throughout the paper, the online source of the annotation data set, the human genome assembly for which annotation was available, references, and comments about preprocessing of the annotation data, respectively.
(PDF)

Table S8 **RT-qPCR primers.** LncRNA expression was validated by RT-qPCR with primers designed using Primer3 (v0.4.0) with default parameters.
(PDF)

Methods S1 **Description of genomic annotation categories.** Detailed description of all genomic annotation categories used to investigate the genomic location of DE-probes.
(PDF)
