# Supplementary figures and images for: Long Non-Coding RNAs Differentially Expressed between Normal versus Primary Breast Tumor Tissues Disclose Converse Changes to Breast Cancer-Related Protein-Coding Genes
Source: PLoS One. 2014 Sep 29;9(9):e106076. doi: 10.1371/journal.pone.0106076 (PMC4180073; doi:10.1371/journal.pone.0106076)

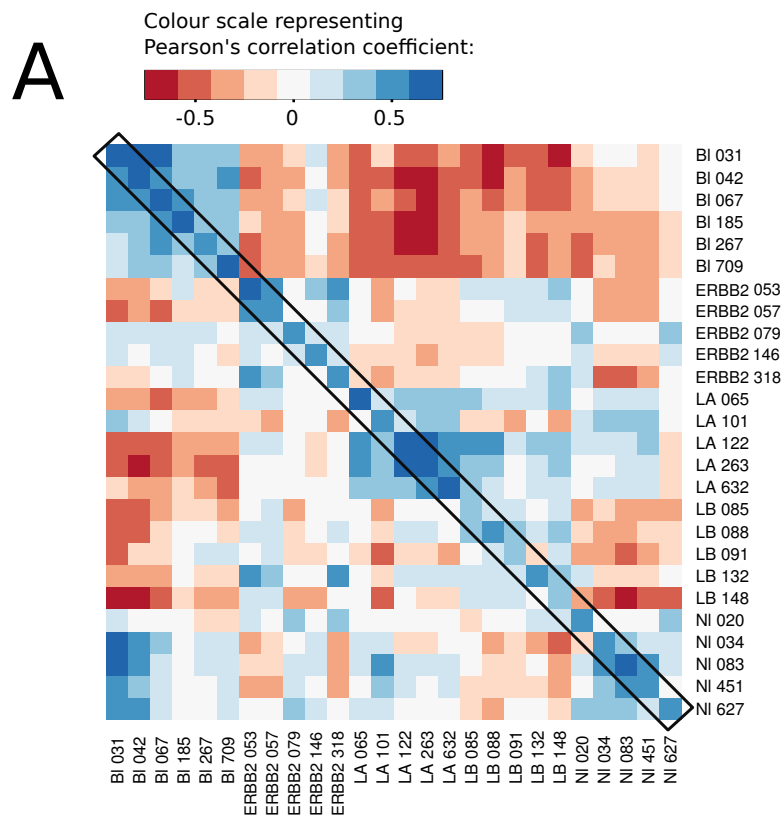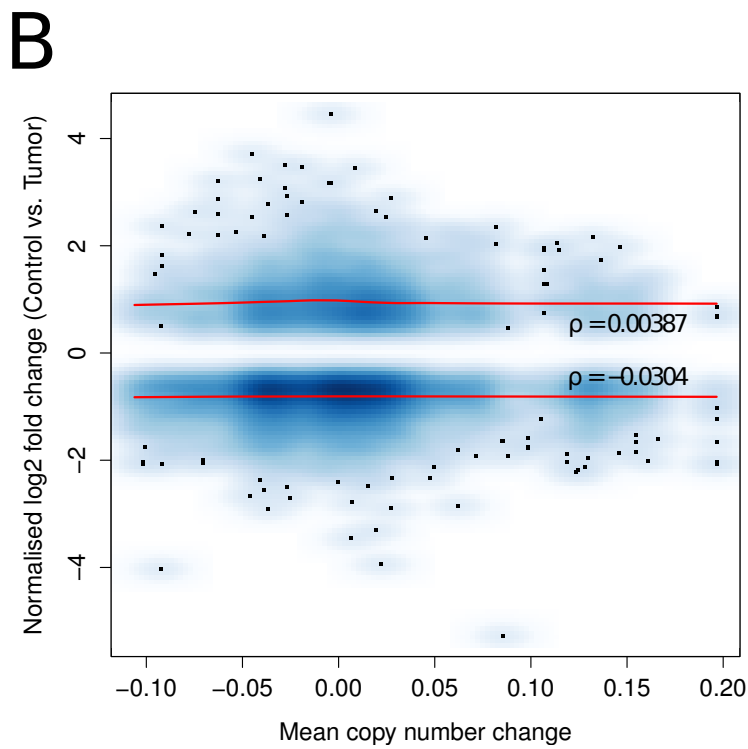

Supplement: Figure S1 — Expression changes recovered mRNA based subtype classification, and were independent of copy number changes. (A.) Heatmap of Pearson's correlation coefficients of expression levels of the protein-coding genes that define the five known mRNA subclasses of breast cancer on the custom microrarray compared to expression levels of these genes in the same set of tissue samples on Agilent 4×44k arrays [28], [44]. Correlation was calculated for 44 of the 50 protein-coding genes defining the mRNA expression breast cancer subclassification. The remaining 6 genes were not represented on the microarray due to the requirement, that at least one probe per gene must be mapped to a unique genomic locus (hg19). PAM50 gene expression is highly correlated between same tumor samples on the two different expression arrays. Abbreviations LA, LB, Bl, and Nl indicate Luminal A, Luminal B, Basal-like, and Normal-like tumor subtypes, respectively. (B.) Smoothed scatterplot of expression changes and copy number variations for non-coding DE-probes regulated between normal and tumor samples (). The portion of probes with similar expression changes and similar copy number variation is reflected by different blue shades. Black dots mark extreme values, and red lines correspond to average intensities for positive and negative expression changes. Spearman's correlation coefficients of and , respectively, indicate that the contribution of the copy number changes have only marginal effect on the expression variation of non-coding regions. (PDF) [file pone.0106076.s001.pdf]

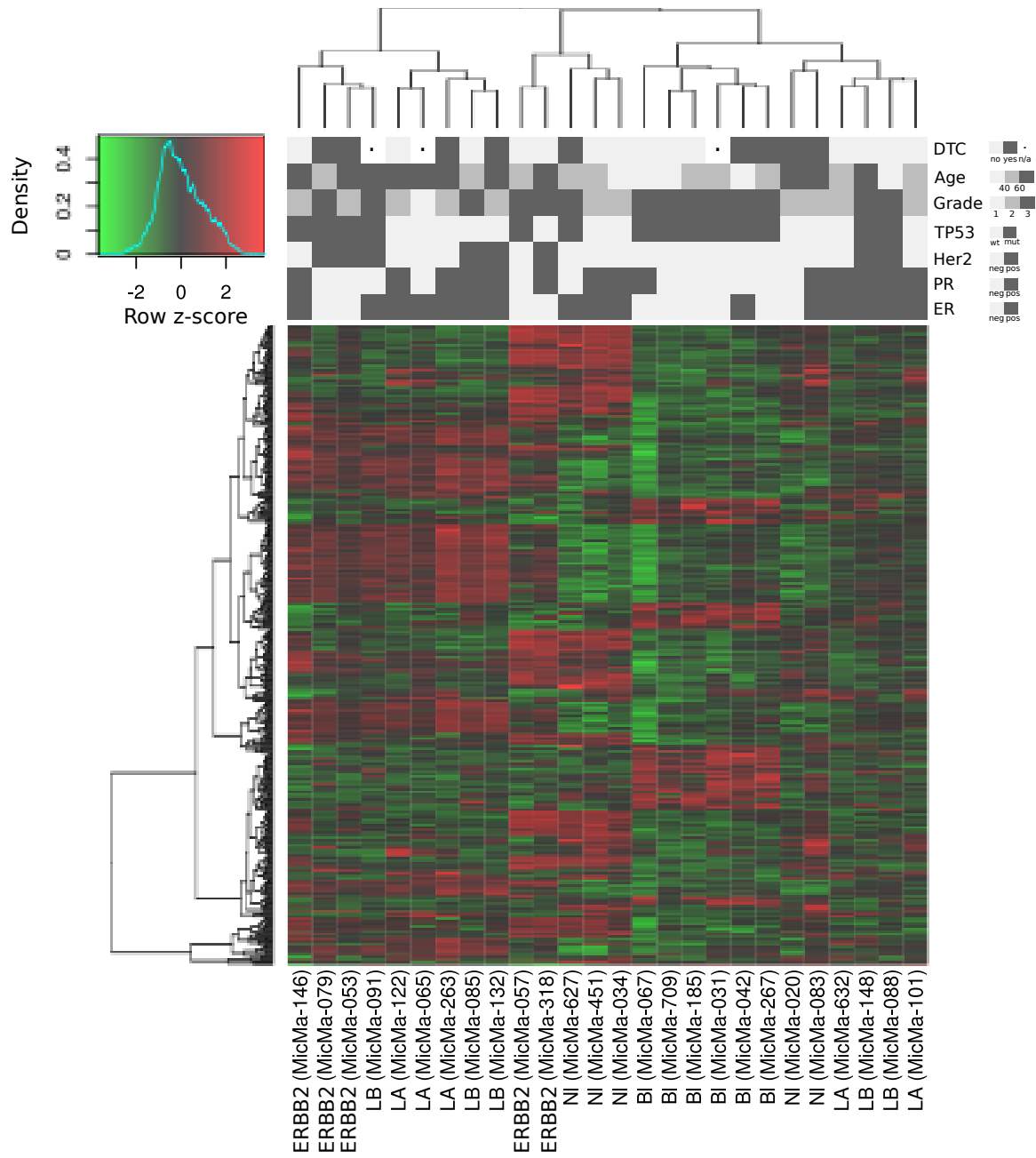

Supplement: Figure S2 — Differential expression of non-coding probes in breast tumor. Heatmap of non-coding probes with significant expression variation between molecular tumor subtypes (F-test with moderated residual mean squares – limma R library, ). Clinical data indicate disseminated tumor cell status (DTC, disseminated tumor cells detected, no = not detected); age at onset (Age); histological grade 1, 2 or 3 (Grade); TP53 mutational status (TP53, wild-type and mutated); status of epidermal growth factor receptor 2 (Her2, Her2 negative, Her2 positive); status of progesterone receptor (PR, PR negative, PR positive); and status of estrogene receptor (ER, ER negative, ER positive). (PDF) [file pone.0106076.s002.pdf]

A Protein-coding probes

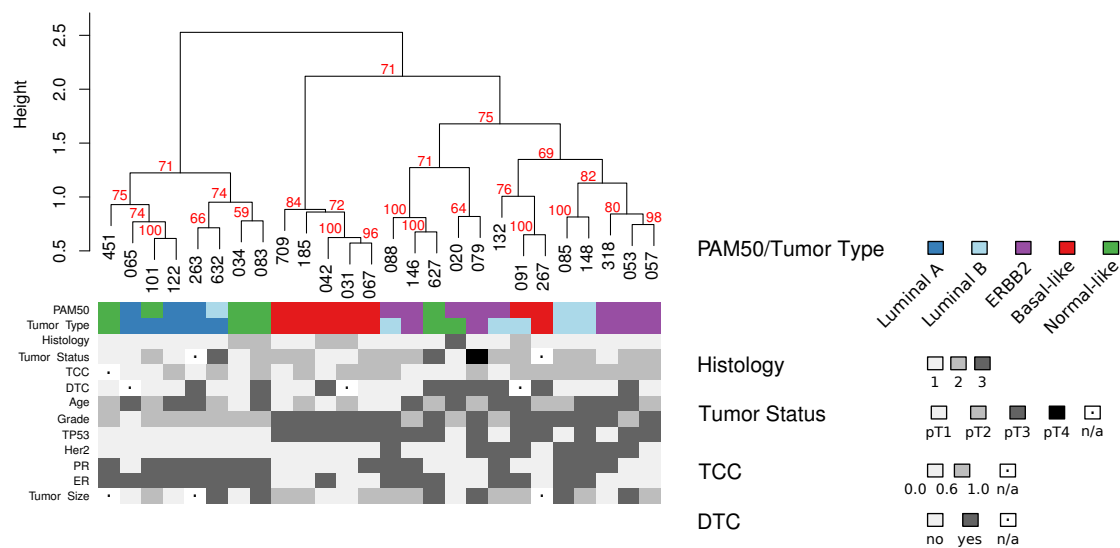

B Non-coding probes

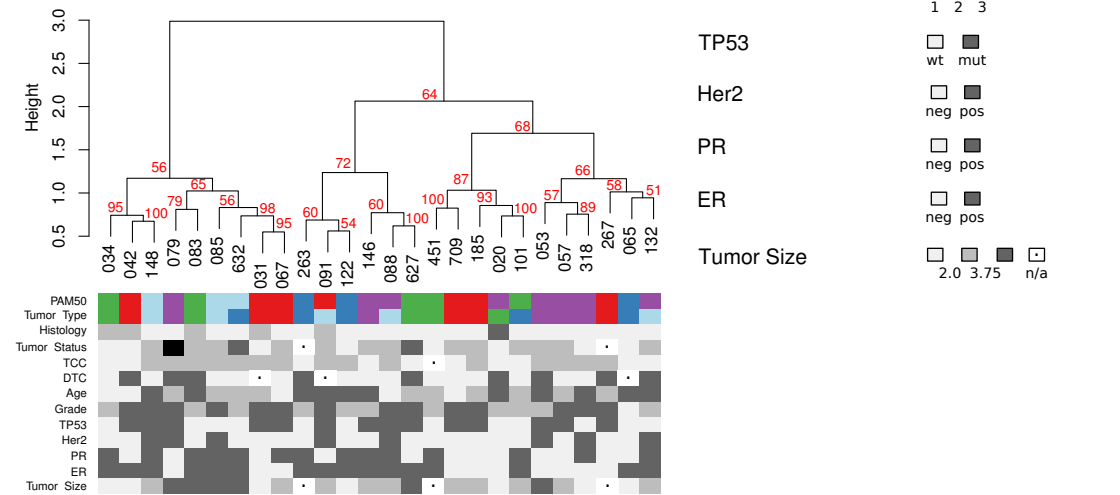

Supplement: Figure S3 — Unsupervised clustering of tumor samples. Hierarchical clustering of probes passing unspecific filtering, i.e. between tumor samples and expression above the background in at least four arrays. (A.) Hierarchical cluster tree of probes located in exons of protein-coding genes (), and (B.) of non-coding probes (). Variance within clusters was minimized by applying Ward's method on scaled intensities of probes, and correlation was used as distance function. Uncertainty of clusters was assessed by bootstrapping with 10,000 iterations (R package pvclust). Red numbers indicate cluster reliability in percent, here with being the significance level to reject the null hypothesis that the cluster is not present in the data. Variation explained by array processing batches was removed prior to clustering (R package limma – removeBatchEffect) in order to receive a clustering of samples which is solely based on biological variation. Detailed description of clinical, pathological and immunohistochemical data of presented tumor samples is provided in caption of Table S1. (PDF) [file pone.0106076.s003.pdf]

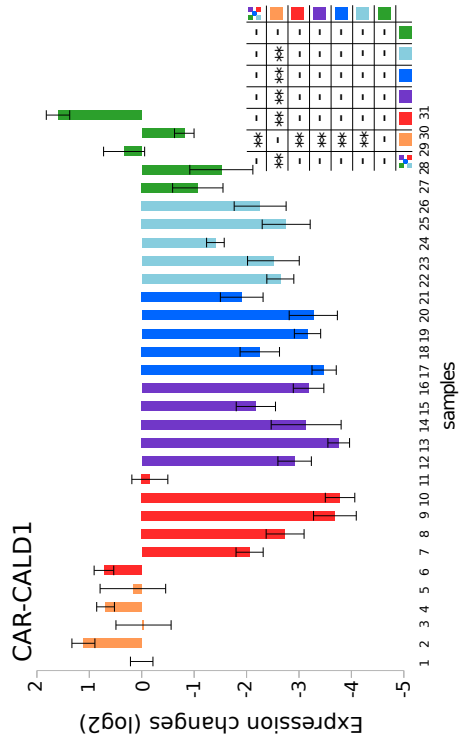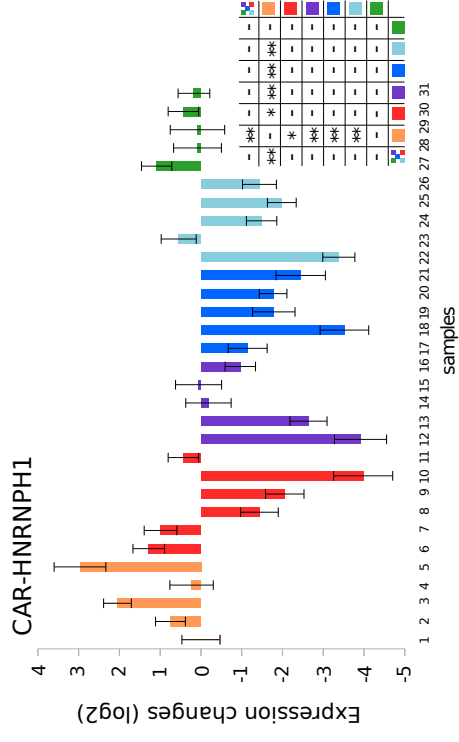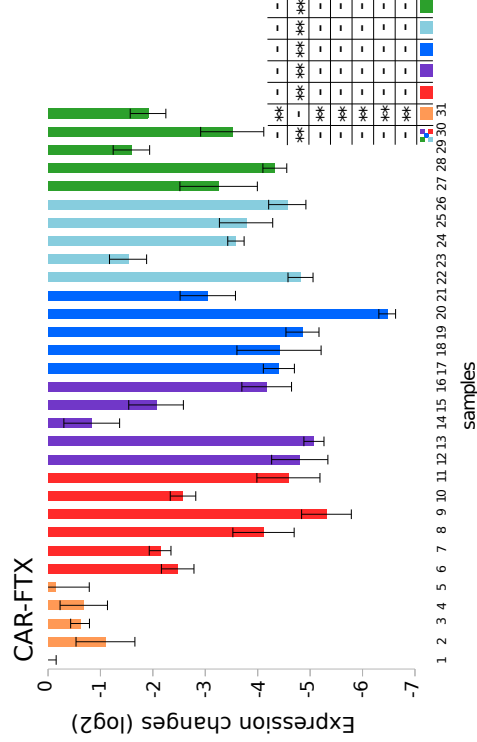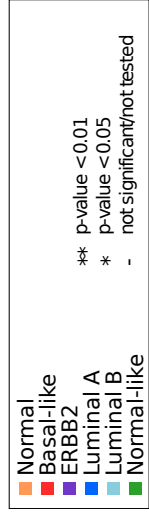

Supplement: Figure S5 — RT-qPCR validation of differentially expressed chromatin-associated lncRNAs. Subsequent analysis of three chromatin-associated lncRNAs (CARs, Table S5) [27] chosen for validation. Validation was performed using all original RNA samples by RT-qPCR. Plots for the chromatin-associated lncRNAs CAR-CALD1 (spanning intron of CALD1 mRNA), CAR-HNRNPH1 (spanning introns and exons of HNRNPH1 mRNA) and CAR-FTX (spanning introns and exons of lincRNA FTX) depict changes in expression (log2 scale). Sample types are represented by different colours: normal breast tissue (yellow); Luminal A subtype (dark blue); normal-like samples (green); the basal-like subtype (red); the ERBB2 samples (purple) and the Luminal B subtype (light blue). The 2D matrix represents the p-value after testing for the different hypotheses (p-value0.01 = **; p-value0.05 = *). (PDF) [file pone.0106076.s005.pdf]

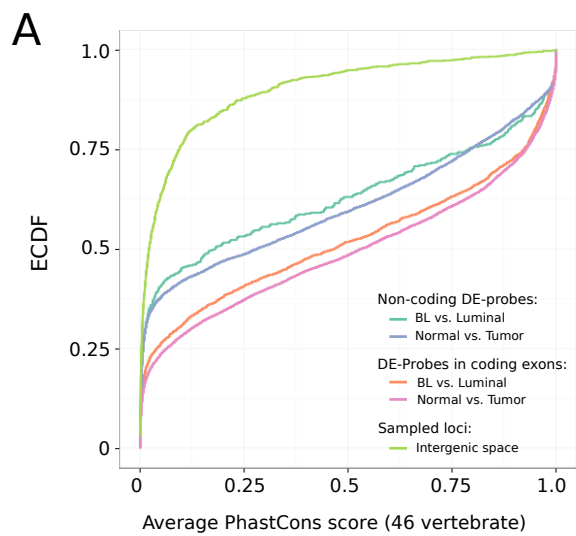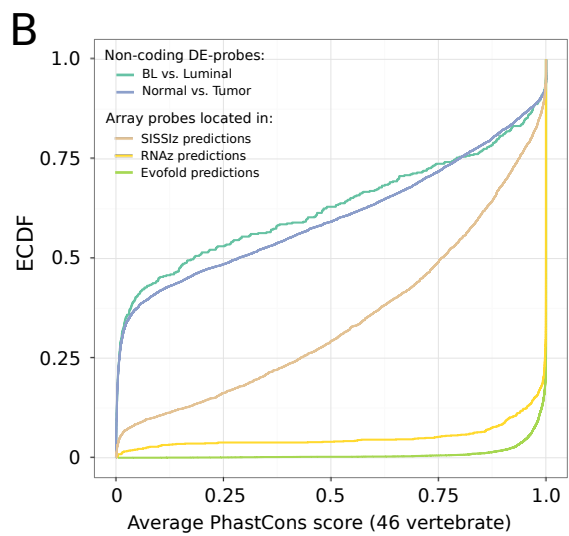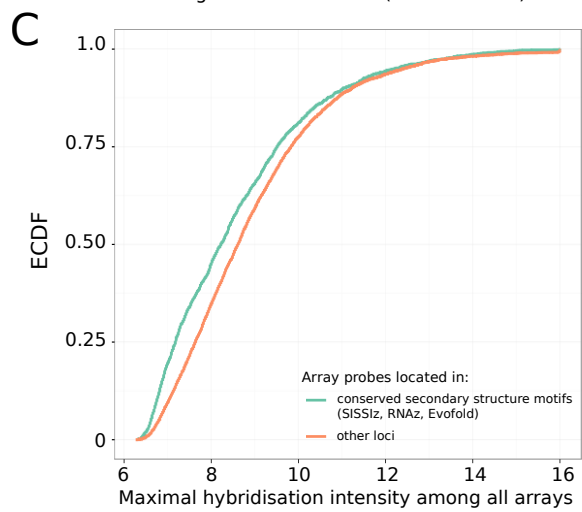

Supplement: Figure S6 — Sequence conservation and hybridisation intensities. Empirical cumulative distributions (ECDF) of average PhastCons scores of DE-probes (Normal vs. Tumor with , Basal-like vs. Luminal tumors with ) either compared to neutral evolving sequences preserving length distribution of coding exons (A.) or to array probes located in genomic loci with conserved secondary structures – RNAz [57], [58], SISSIz [53], [57], and Evofold [59] (B.). (C.) ECDF of maximal microarray hybridisation intensities of probes located in loci with conserved secondary structure motifs compared to all remaining probes on the custom microarray. (PDF) [file pone.0106076.s006.pdf]

**A**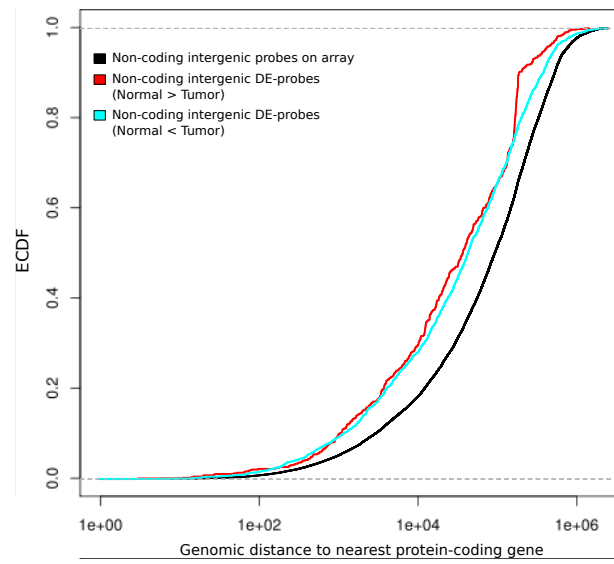**B**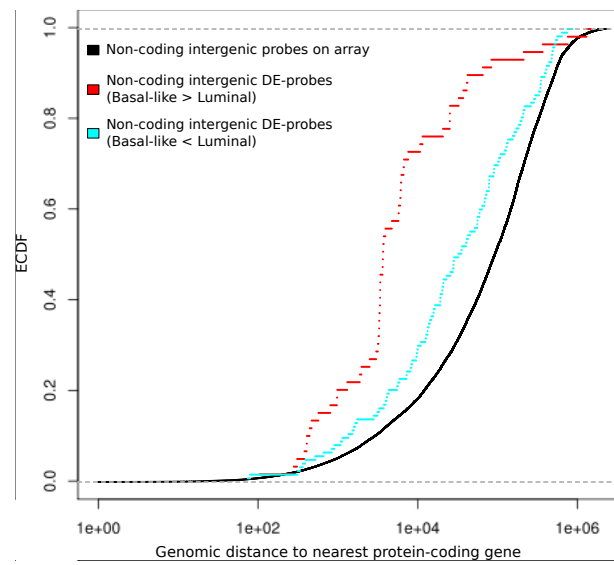

Supplement: Figure S7 — Genomic distance of intergenic non-coding DE-probes to protein-coding genes. Empirical cumulative distribution function (ECDF) of genomic distances of intergenic non-coding probes either significantly differentially expressed between tumor and normal samples (, A.) or between Basal-like and Luminal tumors (, B.) to their nearest protein-coding gene (Gencode v12), not taking the reading direction into account. (PDF) [file pone.0106076.s007.pdf]
