# Supplementary material for: Long Non-Coding RNAs Differentially Expressed between Normal versus Primary Breast Tumor Tissues Disclose Converse Changes to Breast Cancer-Related Protein-Coding Genes
Source: PLoS One. 2014 Sep 29;9(9):e106076. doi: 10.1371/journal.pone.0106076 (PMC4180073; doi:10.1371/journal.pone.0106076)
Supplement: Figure S4 — Differential expression of lncRNAs. Heatmap of lncRNA (Gencode v12) expression changes between normal and tumor tissue. For each lncRNA and patient sample, the median expression of all significantly differentially expressed probes () located in exons of the lncRNA is depicted. Clinical data indicate disseminated tumor cell status (DTC, disseminated tumor cells detected, not detected); age at onset (Age); histological grade 1, 2 or 3 (Grade); TP53 mutational status (TP53, wild-type and mutated); status of epidermal growth factor receptor 2 (Her2, Her2 negative, Her2 positive); status of progesterone receptor (PR, PR negative, PR positive); and status of estrogene receptor (ER, ER negative, ER positive). (PDF) [file pone.0106076.s004.pdf]

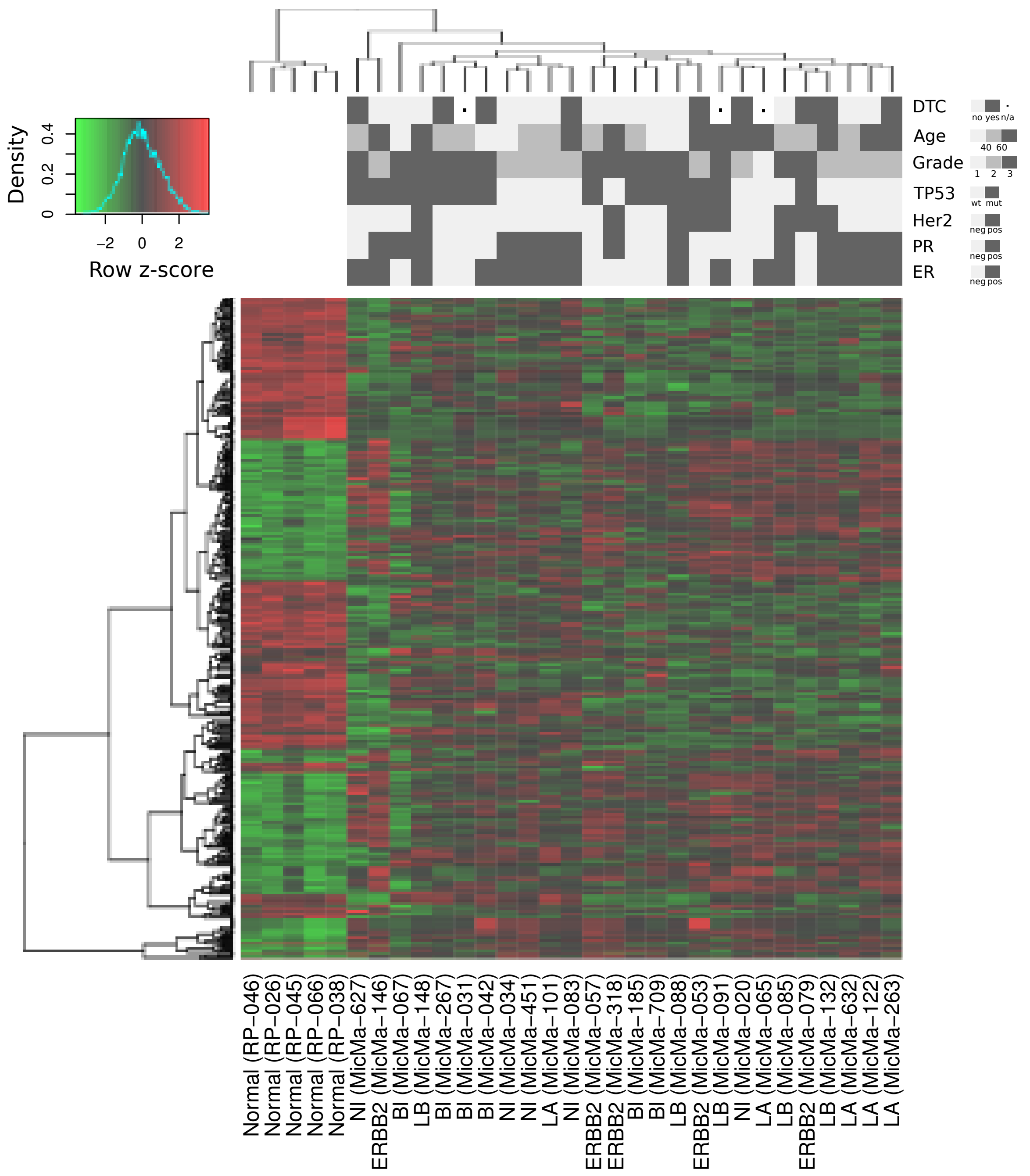

Density
0.4
0.2
0
−2
0
2
Row z-score
DTC
Age
Grade
TP53
Her2
PR
ER
no yes n/a
40 60
1 2 3
wt mut
neg pos
neg pos
neg pos
Normal (RP−046)
Normal (RP−026)
Normal (RP−045)
Normal (RP−066)
Normal (RP−038)
NI (MicMa−627)
ERBB2 (MicMa−146)
BI (MicMa−067)
LB (MicMa−148)
BI (MicMa−267)
BI (MicMa−031)
BI (MicMa−042)
NI (MicMa−034)
NI (MicMa−451)
LA (MicMa−101)
NI (MicMa−083)
ERBB2 (MicMa−057)
ERBB2 (MicMa−318)
BI (MicMa−185)
BI (MicMa−709)
LB (MicMa−088)
ERBB2 (MicMa−053)
LB (MicMa−091)
NI (MicMa−020)
LA (MicMa−065)
LB (MicMa−085)
ERBB2 (MicMa−079)
LB (MicMa−132)
LA (MicMa−632)
LA (MicMa−122)
LA (MicMa−263)
