# Supplementary material for: Long Non-Coding RNAs Differentially Expressed between Normal versus Primary Breast Tumor Tissues Disclose Converse Changes to Breast Cancer-Related Protein-Coding Genes
Source: PLoS One. 2014 Sep 29;9(9):e106076. doi: 10.1371/journal.pone.0106076 (PMC4180073; doi:10.1371/journal.pone.0106076)
Supplement: Table S1 — Clinical, pathological, and immunohistochemical data of presented breast tumor samples. Column headings indicate sample identifier (Sample ID); Age at onset (Age, years with one decimal); tumor cell content in percentage (TCC, , n/a if not available); tumor size in cm (Tumor Size); status of breast tumor (Tumor Status, , [], [], [], [infiltrating skin or thoracic wall], [Carcinoma in situ], [no detected primary tumor], not available); histology (Histology, [invasive ductal carcinoma], [invasive lobular carcinoma], , [ductal carcinoma in situ]); histological grade 1, 2 or 3 (Grade); status of estrogene receptor (ER status, , ); status of progesterone (PR status, , ); HER2 status – combination of IHC and FISH (HER2 combined, [either or ], [either or ], n/amissing); TP53 mutational status (TP53 status, , ); disseminated tumor cell status (DTC status, , ); PAM50-based tumor subtype (PAM50 subtype, , , , , ) and 44k mRNA expression-based subtype (Tumor subtype, , , , , ). Details of DTC detection are further described in Wiedswang et al. 2003 [80]. (PDF) [file pone.0106076.s008.pdf]

												Subtype	
Sample ID	Age	TCC	Tumor Size	Tumor Status	Histology	Grade	ER Status	PR Status	HER2 combined	TP53 Status	DTC Status	PAM50	Tumor
MicMa020	64.0	0.3	1.0	1	3	2	0	0	0	0	1	3	5
MicMa031	43.0	0.9	1.0	1	2	3	0	0	0	1	n/a	4	4
MicMa034	39.0	0.2	1.3	1	2	2	1	1	0	0	0	5	5
MicMa042	36.0	0.6	1.4	1	2	3	1	0	0	1	1	4	4
MicMa053	63.1	1.0	4.0	2	1	2	0	0	1	1	1	3	3
MicMa057	43.1	1.0	2.5	2	1	3	0	0	0	1	0	3	3
MicMa065	73.2	0.6	2.0	1	1	1	1	0	0	0	n/a	1	1
MicMa067	39.2	1.0	2.7	2	1	3	0	1	0	1	0	4	4
MicMa079	47.3	1.0	4.0	4	1	3	0	0	1	1	1	3	3
MicMa083	69.3	0.7	4.0	2	2	2	1	1	0	0	1	5	5
MicMa085	46.3	0.9	4.5	2	1	3	1	1	1	0	0	2	2
MicMa088	33.3	0.9	3.5	2	1	3	1	1	1	1	0	3	2
MicMa091	65.4	1.0	3.0	2	2	3	1	0	1	1	n/a	4	2
MicMa101	42.4	0.6	2.4	2	1	2	1	1	0	0	0	5	1
MicMa122	76.5	0.9	1.5	1	1	2	1	1	0	0	0	1	1
MicMa132	69.6	0.2	3.0	2	1	2	1	1	1	0	1	3	2
MicMa146	72.6	0.6	3.5	2	1	2	1	1	0	1	0	3	3
MicMa148	67.6	1.0	3.2	2	1	3	1	1	1	1	0	2	2
MicMa185	54.7	0.8	2.1	2	1	3	0	0	0	1	0	4	4
MicMa263	71.0	0.5	n/a	n/a	1	2	1	1	0	0	1	1	1
MicMa267	51.0	1.0	n/a	n/a	1	3	0	0	0	1	1	4	4
MicMa318	72.3	0.8	2.0	1	1	3	0	1	1	0	0	3	3
MicMa451	50.7	n/a	n/a	1	1	2	1	1	0	0	0	5	5

												Subtype	
Sample ID	Age	TCC	Tumor Size	Tumor Status	Histology	Grade	ER Status	PR Status	HER2 combined	TP53 Status	DTC Status	PAM50	Tumor
MicMa627	49.2	0.6	5.5	3	1	3	1	0	0	1	1	5	5
MicMa632	56.3	0.9	6.0	3	1	2	1	1	0	0	0	2	1
MicMa709	32.5	0.6	2.5	2	1	3	0	0	0	1	0	4	4
