# Supplementary material for: Long Non-Coding RNAs Differentially Expressed between Normal versus Primary Breast Tumor Tissues Disclose Converse Changes to Breast Cancer-Related Protein-Coding Genes
Source: PLoS One. 2014 Sep 29;9(9):e106076. doi: 10.1371/journal.pone.0106076 (PMC4180073; doi:10.1371/journal.pone.0106076)
Supplement: Table S2 — KEGG pathway enrichment analysis for protein-coding genes significantly differentially expressed (Normal versus Tumor). Most enriched KEGG pathways () of genes significantly differentially expressed between normal and tumor samples (Gencode release v12, ). Column headings indicate ID of KEGG pathway (ID), significance of enrichment (P-value), odds ratios (Odds ratio), expected number of genes associated with tested pathway (Exp. count), number of significantly differentially expressed genes associated with this pathway (Count), number of genes from the gene universe that are annotated in that pathway (Size), name of the pathway (Pathway Name), and a list of genes which are regulated in that pathway and were significantly differentially expressed. Analysis was done by using the Bioconductor GOstats package. Mapping of genes to Entrez IDs is based on the NCBI gene information table (version: July 1, 2012). Significance of enrichment was assessed by a one-sided hypergeometric test where the universe contains all genes of the custom microarray which passed unspecific filtering (Materials and Methods). (PDF) [file pone.0106076.s009.pdf]

	ID	P-value	Odds ratio	Exp. count	Count	Size	Pathway Name	Genes
Normal > Tumor								
1	04510	2.425E-07	2.599	36.387	63	125	Focal adhesion	ACTN4, ACTN1, BIRC2, BRAF, CAV1, CAV2, CCND2, CCND3, COL4A1, COL6A1, COL6A2, CRK, CTNNB1, EGF, FLNA, FLNB, FLNC, FLT4, ARHGAP35, GSK3B, IGF1, IGF1R, ILK, ITGA2, ITGA9, ITGB1, ITGB4, ITGB8, JUN, KDR, LAMA3, LAMA4, LAMB1, LAMB3, LAMC1, LAMC2, MET, MYLK, PPP1R12A, PDGFRA, PDGFRB, PIK3R1, PPP1CA, PPP1CC, MAPK10, PTEN, PXN, RAC1, RAC2, SOS1, THBS3, THBS4, TLN1, VCL, VEGFC, ROCK2, AKT3, MYL9, SHC2, PDGFC, TNN, PDGFD, TLN2
2	04360	8.701E-06	2.708	24.161	43	83	Axon guidance	ABL1, CFL2, DPYSL2, EFNA5, EFNB2, EFNB3, EPHA4, EPHB1, EPHB3, EPHB4, FES, GNAI2, GSK3B, ITGB1, ABLIM1, LIMK2, MET, NCK1, NFATC3, NFATC4, PLXNB1, PPP3CA, PPP3CB, RAC1, RAC2, RGS3, CXCL12, SLIT3, NCK2, SEMA5A, SLIT2, NTN1, ROCK2, SRGAP3, SEMA4D, SEMA4B, NFAT5, ARHGEF12, PLXNB2, NTN4, ROBO3, SEMA6D, SEMA3D
3	04520	2.378E-05	3.545	13.391	27	46	Adherens junction	ACTN4, ACTN1, CSNK2A1, CTNNB1, CTNND1, EP300, FGFR1, IGF1R, INSR, SMAD2, MET, MLLT4, PTPRF, PTPRM, PVRL1, RAC1, RAC2, SNAI2, TCF7, TCF7L2, TGFBR2, TJP1, VCL, IQGAP1, SORBS1, PARD3, PVRL4
4	04310	8.783E-05	2.231	29.110	47	100	Wnt signaling pathway	APC, CAMK2B, CAMK2G, CCND2, CCND3, CSNK1A1, CSNK1E, CSNK2A1, CTBP1, CTNNB1, DVL1, DVL3, EP300, FZD2, GSK3B, JUN, LRP6, LRP5, SMAD2, MYC, NFATC3, NFATC4, PPARD, PPP2R1B, PPP2R5C, PPP3CA, PPP3CB, PRKACB, MAPK10, PSEN1, RAC1, RAC2, SFRP1, TCF7, TCF7L2, TP53, FZD5, AXIN2, FZD4, FZD7, BTRC, ROCK2, APC2, NFAT5, DAAM2, WNT5B, PRICKLE2

	ID	P-value	Odds ratio	Exp. count	Count	Size	Pathway Name	Genes
5	05200	1.994E-04	1.705	63.168	87	217	Pathways in cancer	ABL1, APC, BIRC2, FAS, ARNT, BRAF, CASP9, RUNX1, RUNX1T1, CBL, CBLB, CDK6, CEBPA, COL4A1, CRK, CTBP1, CTNNB1, DVL1, DVL3, EGF, EP300, EPAS1, ETS1, FGF1, FGF2, FGFR1, FGFR2, FOXO1, FOS, MTOR, FZD2, GLI3, GSK3B, IGF1, IGF1R, IKBKB, ITGA2, ITGB1, JUN, JUP, KIT, LAMA3, LAMA4, LAMB1, LAMB3, LAMC1, LAMC2, SMAD2, MET, MYC, PDGFRA, PDGFRB, PIK3R1, PLCG1, PLD1, PPARD, PPARG, MAPK10, PTCH1, PTEN, RAC1, RAC2, RXRB, RXRG, SMO, SOS1, STAT3, STAT5B, TCF7, TCF7L2, TGFBR2, TP53, VEGFC, ZBTB16, FZD5, AXIN2, FZD4, FZD7, PIAS2, AKT3, APC2, PIAS3, DAPK2, APPL1, SUFU, EGLN1, WNT5B
6	04810	2.539E-04	1.970	35.805	54	123	Regulation of actin cytoskeleton	ACTN4, ACTN1, APC, BDKRB2, BRAF, CFL2, CRK, DIAPH2, EGF, FGF1, FGF2, FGFR1, FGFR2, FGFR4, ARHGAP35, GSN, ITGA2, ITGA9, ITGB1, ITGB4, ITGB8, LIMK2, MSN, MYH9, MYH10, MYLK, PPP1R12A, PDGFRA, PDGFRB, PIK3R1, PPP1CA, PPP1CC, PXN, RAC1, RAC2, RRAS, SOS1, TMSB4X, VCL, PIP4K2B, IQGAP1, ARHGEF7, ARHGEF6, ROCK2, ARPC4, ABI2, APC2, MYL9, MRAS, CYFIP1, ARHGEF12, SSH1, PDGFC, PDGFD
7	05217	5.569E-04	3.360	9.606	19	33	Basal cell carcinoma	APC, CTNNB1, DVL1, DVL3, FZD2, GLI3, GSK3B, PTCH1, SMO, TCF7, TCF7L2, TP53, FZD5, AXIN2, FZD4, FZD7, APC2, SUFU, WNT5B
8	00310	1.135E-03	3.179	9.315	18	32	Lysine degradation	ACAT1, ALDH2, ALDH7A1, GCDH, HADHA, OGDH, SETMAR, BBOX1, PLOD3, SETDB1, EHMT2, SETD2, SUV420H1, WHSC1L1, ASH1L, NSD1, EHMT1, SETD7
9	03040	1.698E-03	2.106	20.959	33	72	Spliceosome	HNRNPK, NHP2L1, PCBP1, PLRG1, SRSF1, SRSF2, SRSF3, SRSF4, SRSF5, TRA2B, SNRNP70, DDX39B, DHX16, SART1, SNRNP40, DHX38, SF3A1, CHERP, SLU7, PRPF8, TCERG1, DDX42, U2AF2, NCBP2, ACIN1, U2SURP, SF3B1, SYF2, RBMX, PRPF38B, XAB2, RBM25, PRPF38A

	ID	P-value	Odds ratio	Exp. count	Count	Size	Pathway Name	Genes
10	03320	5.359E-03	2.621	9.606	17	33	PPAR signaling pathway	CPT1A, ILK, LPL, PLIN1, PLTP, PPARA, PPARD, PPARG, RXRB, RXRG, SCD, UBC, ADIPOQ, NR1H3, SORBS1, ANGPTL4, SLC27A1
11	04530	5.779E-03	1.923	20.668	31	71	Tight junction	ACTN4, ACTN1, CSNK2A1, CTNNB1, EPB41L1, EPB41L2, GNAI2, MLLT4, MYH9, MYH10, MYH11, CLDN11, PPP2R1B, PRKCH, PTEN, RRAS, SPTAN1, TJP1, CLDN5, VAPA, MAGI1, TJP2, AKT3, MYL9, EXOC3, MRAS, PPP2R2D, ASH1L, PARD3, JAM3, AMOTL1
12	05213	1.567E-02	2.204	10.480	17	36	Endometrial cancer	APC, BRAF, CASP9, CTNNB1, EGF, GSK3B, ILK, MYC, PIK3R1, PTEN, SOS1, TCF7, TCF7L2, TP53, AXIN2, AKT3, APC2
13	05215	1.578E-02	1.836	17.757	26	61	Prostate cancer	BRAF, CASP9, CTNNB1, EGF, EP300, FGFR1, FGFR2, FOXO1, MTOR, GSK3B, IGF1, IGF1R, IKBKB, PDGFRA, PDGFRB, PIK3R1, PTEN, SOS1, TCF7, TCF7L2, TP53, CREB5, AKT3, PDGFC, CREB3L2, PDGFD
14	04920	2.134E-02	2.093	10.771	17	37	Adipocytokine signaling pathway	CPT1A, MTOR, IKBKB, IRS1, PPARA, PRKAB1, MAPK10, RXRB, RXRG, STAT3, IRS2, SOCS3, ADIPOQ, AKT3, CAMKK2, ADIPOR2, CAMKK1
15	05221	3.009E-02	1.926	11.935	18	41	Acute myeloid leukemia	BRAF, RUNX1, RUNX1T1, CEBPA, MTOR, IKBKB, JUP, KIT, MYC, PIK3R1, PPARD, SOS1, STAT3, STAT5B, TCF7, TCF7L2, ZBTB16, AKT3
16	05210	3.386E-02	1.784	14.555	21	50	Colorectal cancer	APC, BRAF, CASP9, CTNNB1, FOS, GSK3B, JUN, SMAD2, MYC, PIK3R1, MAPK10, RAC1, RAC2, TCF7, TCF7L2, TGFBR2, TP53, AXIN2, AKT3, APC2, APPL1
17	04010	3.486E-02	1.388	46.285	57	159	MAPK signaling pathway	FAS, BRAF, CRK, DUSP1, DUSP3, DUSP6, EGF, ELK4, FGF1, FGF2, FGFR1, FGFR2, FGFR4, FLNA, FLNB, FLNC, FOS, NR4A1, IKBKB, JUN, JUND, MEF2C, MAP3K4, MYC, NFATC4, NTF3, NTRK2, PDGFRA, PDGFRB, PPM1A, PPM1B, PPP3CA, PPP3CB, PRKACB, MAPK10, MAP2K7, RAC1, RAC2, RASA2, RRAS, SOS1, SRF, TGFBR2, TP53, MAPKAPK5, JMJD7-PLA2G4B, RPS6KA4, TAOK2, AKT3, RASGRP2, MRAS, TAB2, MAPK8IP3, TAOK3, DUSP22, TAOK1, CACNG7

	ID	P-value	Odds ratio	Exp. count	Count	Size	Pathway Name	Genes
18	04340	3.828E-02	2.129	8.151	13	28	Hedgehog signaling pathway	BMP6, CSNK1A1, CSNK1D, CSNK1E, GAS1, GLI3, GSK3B, PRKACB, PTCH1, SMO, BTRC, SUFU, WNT5B
19	04512	4.230E-02	1.723	14.846	21	51	ECM-receptor interaction	CD47, COL4A1, COL6A1, COL6A2, DAG1, ITGA2, ITGA9, ITGB1, ITGB4, ITGB8, LAMA3, LAMA4, LAMB1, LAMB3, LAMC1, LAMC2, THBS3, THBS4, SDC3, TNN, AGRN
20	04722	4.506E-02	1.509	25.326	33	87	Neurotrophin signaling pathway	ABL1, BRAF, CALM1, CAMK2B, CAMK2G, CRK, GAB1, GSK3B, IKBKB, IRS1, JUN, NGFR, NTF3, NTRK2, PIK3R1, PLCG1, MAPK10, MAP2K7, PSEN1, RAC1, SOS1, TP53, YWHAB, YWHAG, IRS2, RPS6KA4, AKT3, SH2B3, ZNF274, PRDM4, SHC2, SH2B1, KIDINS220
21	04630	4.524E-02	1.585	20.086	27	69	Jak-STAT signaling pathway	CBL, CBLB, CCND2, CCND3, EP300, EPOR, GHR, IF-NAR1, IFNGR1, LIFR, MYC, PIK3R1, SOS1, STAT2, STAT3, STAT5B, STAT6, TYK2, SOCS2, SOCS3, PIAS2, AKT3, SPRY1, SPRY2, PIAS3, IL20RA, SOCS4
22	04144	4.834E-02	1.423	33.476	42	115	Endocytosis	ADRBK2, AP2A2, CAV1, CAV2, CBL, CBLB, DAB2, DNM1, DNM2, EGF, EPS15, FGFR2, FGFR4, FOLR1, FOLR2, IGF1R, KDR, KIT, SMAD2, MET, PDGFRA, PLD1, TGFBR2, GIT2, IQSEC1, VPS45, RAB11FIP2, NEDD4L, PSD4, VPS4A, CHMP2A, SH3KBP1, EHD4, ASAP1, VPS36, ARFGAP1, PARD3, SH3GLB2, VPS37B, RUFY1, FAM125B, ARAP1
23	05412	4.915E-02	1.770	12.517	18	43	Arrhythmogenic right ventricular cardiomyopathy (ARVC)	ACTN4, ACTN1, CTNNB1, DAG1, DMD, DSG2, ITGA2, ITGA9, ITGB1, ITGB4, ITGB8, JUP, LMNA, SGCB, SGCD, TCF7, TCF7L2, CACNG7
							Normal < Tumor	
1	05322	5.945E-07	3.376	15.964	35	75	Systemic lupus erythematosus	C2, HIST1H2AE, H2AFX, H2AFZ, HIST1H2BB, HLA-DQB1, HIST1H2AK, HIST1H2AL, HIST1H2AC, HIST1H2AB, HIST1H2AM, HIST1H2BG, HIST1H2BL, HIST1H2BM, HIST1H2BH, HIST1H2BI, HIST1H2BC, HIST1H2BO, HIST1H3A, HIST1H3D, HIST1H3C, HIST1H3E, HIST1H3I, HIST1H3G, HIST1H3J, HIST1H3H, HIST1H3B, HIST1H4A, HIST1H4F, HIST1H4L, HIST1H3F, HIST1H2AG, HIST1H2BJ, H2AFJ, HIST3H2A

	ID	P-value	Odds ratio	Exp. count	Count	Size	Pathway Name	Genes
2	04260	3.025E-05	3.994	8.301	20	39	Cardiac muscle contraction	ACTC1, ATP1A1, ATP1B2, CACNA1C, CACNA1S, CACNB1, COX6B1, COX6C, COX7A2, COX7B, COX8A, MYH6, TNNC1, TNNI3, COX7A2L, CACNA2D2, UQCRQ, CACNG4, UQCR10, CACNG7
3	04110	2.044E-04	2.396	17.667	32	83	Cell cycle	BUB1B, CCNA2, CCNB1, CCNE1, CDK1, CDC6, CDC25A, CDC25B, CDC25C, CDK2, CDKN1A, CDKN2A, E2F1, E2F5, GSK3B, MAD2L1, MCM2, MCM3, MCM4, MDM2, PCNA, PLK1, PRKDC, TTK, YWHAG, SMC1A, PKMYT1, CCNB2, CCNE2, BUB3, ORC6, CCNB3
4	00190	6.830E-04	2.527	12.771	24	60	Oxidative phosphorylation	ATP5G3, ATP6V1A, ATP6V1E1, ATP5O, COX6B1, COX6C, COX7A2, COX7B, COX8A, NDUFA2, NDUFA6, NDUFB2, NDUFB9, NDUFS1, NDUFS8, COX7A2L, ATP5L, UQCRQ, UQCR10, ATP6V1H, NDUFB11, NDUFA12, ATP6V1C2, NDUFS7
5	05012	4.765E-03	2.200	12.132	21	57	Parkinson’s disease	ATP5G3, ATP5O, CASP3, COX6B1, COX6C, COX7A2, COX7B, COX8A, NDUFA2, NDUFA6, NDUFB2, NDUFB9, NDUFS1, NDUFS8, TH, COX7A2L, UQCRQ, UQCR10, NDUFB11, NDUFA12, NDUFS7
6	04914	2.145E-02	1.930	11.281	18	53	Progesterone-mediated oocyte maturation	ARAF, CCNA2, CCNB1, CDK1, CDC25A, CDC25B, CDC25C, CDK2, GNAI2, IGF1, MAD2L1, PLK1, MAPK13, RPS6KA2, PKMYT1, CCNB2, PIK3R5, CCNB3
7	05010	2.682E-02	1.622	19.795	28	93	Alzheimer’s disease	ATP5G3, ATP5O, CACNA1C, CACNA1S, CALM2, CASP3, COX6B1, COX6C, COX7A2, COX7B, COX8A, GSK3B, LRP1, NDUFA2, NDUFA6, NDUFB2, NDUFB9, NDUFS1, NDUFS8, NOS1, COX7A2L, NCSTN, UQCRQ, UQCR10, NDUFB11, PSENEN, NDUFA12, NDUFS7
8	05016	2.682E-02	1.622	19.795	28	93	Huntington’s disease	ATP5G3, ATP5O, BDNF, CASP3, CLTC, COX6B1, COX6C, COX7A2, COX7B, COX8A, NDUFA2, NDUFA6, NDUFB2, NDUFB9, NDUFS1, NDUFS8, POLR2K, TAF4, TGM2, COX7A2L, DCTN2, RCOR1, UQCRQ, UQCR10, NDUFB11, NDUFA12, CREB3L4, NDUFS7

	ID	P-value	Odds ratio	Exp. count	Count	Size	Pathway Name	Genes
9	04114	3.423E-02	1.686	15.112	22	71	Oocyte meiosis	AR, CALM2, CCNB1, CCNE1, CDK1, CDC25C, CDK2, IGF1, MAD2L1, PLK1, PPP2CA, PPP2R5B, PPP2R5D, RPS6KA2, AURKA, YWHAG, SMC1A, PKMYT1, CCNB2, CCNE2, FBXO5, SGOL1
10	04080	3.994E-02	1.549	20.434	28	96	Neuroactive ligand-receptor interaction	TSPO, C5AR1, CHRM1, CHRM2, CHRNE, LTB4R, DRD1, DRD2, DRD3, GABRA1, NPBWR1, GRIA1, GRIK5, GRM2, GRM7, OPRL1, PPYR1, PRSS3, SSTR1, TACR2, THRA, TRHR, TSHB, CYSLTR1, HRH3, NTSR2, GRIN3A, GPR156
