# Supplementary material for: Long Non-Coding RNAs Differentially Expressed between Normal versus Primary Breast Tumor Tissues Disclose Converse Changes to Breast Cancer-Related Protein-Coding Genes
Source: PLoS One. 2014 Sep 29;9(9):e106076. doi: 10.1371/journal.pone.0106076 (PMC4180073; doi:10.1371/journal.pone.0106076)
Supplement: Table S4 — DE-Probes overlap with genomic annotation. Number of DE-Probes significantly differentially expressed between normal and tumor samples () or Basal-like and Luminal tumors (), and overlapping with diverse sets of genomic annotation. Annotation datasets are described in Table S7. Overlaps are calculated by using the Bioconductor genomeIntervals package [94]. The significance of the observed overlap is assessed by calculating odds ratios of observed (DE-Probes) versus expected (all probes on microarray) relative overlaps. Odds ratios are calculated and tested by Fisher's exact test for significant enrichment or depletion (see Materials and Methods). Column heading Annotation indicates annotation datasets for which overlap is computed, and Survey corresponds to the direction of expression variation (either : overlap for probes downregulated in tumor, : overlap for probes upregulated in tumor; or : overlap for probes upregulated in Basal-like tumors, : overlap for probes downregulated in Basal-like tumors). Remaining columns indicate the results (Odds ratio, P-value, and 95% confidence interval for odds ratio − 95%CI) and the data (DE-Probes: number of differentially expressed probes located in annotation, i.e. fraction of overlapping probe nucleotides 0.9, or non-overlapping with annotation, i.e. fraction of overlapping probe nucleotides 0.9; BG: number of array probes located in annotation, i.e. fraction of overlapping probe nucleotides 0.9, or non-overlapping with annotation, i.e. fraction of overlapping probe nucleotides 0.9) of Fisher's exact test. (PDF) [file pone.0106076.s011.pdf]

Annotation	Survey	Fisher's exact test: Odds ratio	P-value	95% CI	overlap: DE-Probes	BG	no overlap: DE-Probes	BG
**Overlap with protein coding genes and intergenic space (all DE-probes)**								
**Normal vs. Tumor**								
CDS (antisense)	Normal > Tumor	0.86	1.87e-02	[0.76, 0.98]	259	6159	9497	194434
	Normal < Tumor	2.06	3.40e-56	[1.90, 2.24]	665	6159	10182	194434
CDS (sense)	Normal > Tumor	3.96	0.00e+00	[3.76, 4.16]	2229	13972	7527	186621
	Normal < Tumor	1.29	1.17e-12	[1.21, 1.38]	957	13972	9890	186621
intergenic	Normal > Tumor	0.27	0.00e+00	[0.25, 0.28]	992	59919	8764	140674
	Normal < Tumor	0.91	5.65e-06	[0.87, 0.95]	3019	59919	7828	140674
introns (antisense)	Normal > Tumor	0.32	9.74e-267	[0.29, 0.34]	739	41391	9017	159202
	Normal < Tumor	0.91	3.06e-04	[0.87, 0.96]	2083	41391	8764	159202
introns (sense)	Normal > Tumor	0.99	5.94e-01	[0.94, 1.04]	1909	39702	7847	160891
	Normal < Tumor	0.72	9.27e-35	[0.68, 0.76]	1640	39702	9207	160891
3′UTRs (antisense)	Normal > Tumor	0.53	2.52e-31	[0.47, 0.60]	300	11280	9456	189313
	Normal < Tumor	1.29	1.97e-10	[1.19, 1.39]	773	11280	10074	189313
3′UTRs (sense)	Normal > Tumor	3.44	0.00e+00	[3.29, 3.60]	3159	24480	6597	176113
	Normal < Tumor	1.17	8.30e-08	[1.10, 1.24]	1515	24480	9332	176113
5′UTRs (antisense)	Normal > Tumor	0.77	6.77e-02	[0.56, 1.02]	48	1288	9708	199305
	Normal < Tumor	2.04	2.69e-13	[1.70, 2.43]	141	1288	10706	199305
5′UTRs (sense)	Normal > Tumor	2.60	1.32e-30	[2.24, 3.01]	209	1673	9547	198920
	Normal < Tumor	1.69	9.51e-09	[1.42, 2.00]	152	1673	10695	198920
**Basal-like vs. Luminal Tumors**								
CDS (antisense)	BL > Luminal	2.49	1.44e-24	[2.12, 2.91]	176	6159	2230	194434
	BL < Luminal	1.66	1.37e-03	[1.21, 2.22]	48	6159	914	194434
CDS (sense)	BL > Luminal	5.97	6.39e-263	[5.46, 6.52]	743	13972	1663	186621
	BL < Luminal	2.99	2.58e-31	[2.52, 3.53]	176	13972	786	186621
intergenic	BL > Luminal	0.12	3.38e-208	[0.10, 0.15]	120	59919	2286	140674
	BL < Luminal	0.52	8.68e-17	[0.44, 0.61]	174	59919	788	140674
introns (antisense)	BL > Luminal	0.22	2.07e-99	[0.18, 0.26]	129	41391	2277	159202
	BL < Luminal	0.49	1.55e-14	[0.39, 0.60]	108	41391	854	159202
introns (sense)	BL > Luminal	0.22	2.39e-95	[0.18, 0.26]	123	39702	2283	160891
	BL < Luminal	0.54	3.90e-11	[0.44, 0.66]	113	39702	849	160891
3′UTRs (antisense)	BL > Luminal	1.17	6.16e-02	[0.99, 1.38]	157	11280	2249	189313
	BL < Luminal	1.10	4.83e-01	[0.83, 1.43]	59	11280	903	189313
3′UTRs (sense)	BL > Luminal	5.20	4.21e-288	[4.79, 5.65]	1010	24480	1396	176113
	BL < Luminal	2.98	5.03e-45	[2.59, 3.44]	282	24480	680	176113
5′UTRs (antisense)	BL > Luminal	0.58	1.20e-01	[0.26, 1.11]	9	1288	2397	199305
	BL < Luminal	1.13	6.83e-01	[0.45, 2.35]	7	1288	955	199305
5′UTRs (sense)	BL > Luminal	1.45	5.48e-02	[0.97, 2.10]	29	1673	2377	198920
	BL < Luminal	1.76	4.76e-02	[0.95, 2.97]	14	1673	948	198920
**Overlap with known non-coding RNAs (non-coding DE-probes)**								
**Normal vs. Tumor**								

		Fisher's exact test			overlap		no overlap	
Annotation	Survey	Odds ratio	P-value	95% CI	DE-Probes	BG	DE-Probes	BG
CARs	Normal > Tumor	5.75	8.22e-95	[4.99, 6.60]	250	2073	2574	122732
	Normal < Tumor	0.84	1.39e-01	[0.67, 1.06]	81	2073	5685	122732
Evofold	Normal > Tumor	0.36	6.00e-22	[0.27, 0.46]	65	7726	2759	117079
	Normal < Tumor	0.32	2.11e-48	[0.27, 0.39]	120	7726	5646	117079
lincRNAs	Normal > Tumor	2.81	1.24e-13	[2.18, 3.57]	73	1167	2751	123638
	Normal < Tumor	0.91	5.74e-01	[0.67, 1.21]	49	1167	5717	123638
lncRNAdb	Normal > Tumor	10.09	3.88e-47	[7.78,12.95]	79	355	2745	124450
	Normal < Tumor	0.61	1.26e-01	[0.29, 1.13]	10	355	5756	124450
lncRNAs (Gencode)	Normal > Tumor	1.81	1.60e-03	[1.24, 2.56]	34	834	2790	123971
	Normal < Tumor	1.38	2.67e-02	[1.02, 1.82]	53	834	5713	123971
PINs	Normal > Tumor	3.69	2.16e-10	[2.55, 5.20]	36	435	2788	124370
	Normal < Tumor	0.70	2.05e-01	[0.38, 1.18]	14	435	5752	124370
RNAz	Normal > Tumor	0.97	1.00e+00	[0.56, 1.57]	17	773	2807	124032
	Normal < Tumor	0.45	4.65e-04	[0.25, 0.73]	16	773	5750	124032
miRNAs	Normal > Tumor	0.94	1.00e+00	[0.30, 2.22]	5	236	2819	124569
	Normal < Tumor	1.19	5.34e-01	[0.63, 2.08]	13	236	5753	124569
snoRNAs or scaRNAs	Normal > Tumor	3.65	4.29e-08	[2.37, 5.42]	27	329	2797	124476
	Normal < Tumor	4.92	2.63e-25	[3.76, 6.36]	74	329	5692	124476
SISSIz	Normal > Tumor	1.01	9.39e-01	[0.73, 1.36]	45	1976	2779	122829
	Normal < Tumor	1.51	1.11e-05	[1.26, 1.80]	137	1976	5629	122829
TINs	Normal > Tumor	2.28	4.41e-14	[1.87, 2.77]	114	2260	2710	122545
	Normal < Tumor	0.80	4.78e-02	[0.64, 1.00]	84	2260	5682	122545
TUCP	Normal > Tumor	4.58	4.02e-13	[3.16, 6.44]	37	361	2787	124444
	Normal < Tumor	0.72	3.12e-01	[0.37, 1.27]	12	361	5754	124444
			Basal-like vs. Luminal Tumors					
CARs	BL > Luminal	0.37	5.31e-01	[0.01, 2.10]	1	2073	162	122732
	BL < Luminal	1.90	6.32e-02	[0.86, 3.70]	9	2073	281	122732
Evofold	BL > Luminal	0.38	4.90e-02	[0.10, 1.00]	4	7726	159	117079
	BL < Luminal	0.60	1.11e-01	[0.29, 1.10]	11	7726	279	117079
lincRNAs	BL > Luminal	1.32	6.67e-01	[0.16, 4.80]	2	1167	161	123638
	BL < Luminal	1.11	7.55e-01	[0.23, 3.30]	3	1167	287	123638
lncRNAdb	BL > Luminal	11.09	1.20e-04	[3.53,26.70]	5	355	158	124450
	BL < Luminal	0.00	1.00e+00	[0.00, 4.50]	0	355	290	124450
lncRNAs (Gencode)	BL > Luminal	6.67	1.31e-04	[2.63,14.10]	7	834	156	123971
	BL < Luminal	1.03	7.21e-01	[0.12, 3.80]	2	834	288	123971
PINs	BL > Luminal	0.00	1.00e+00	[0.00, 6.60]	0	435	163	124370
	BL < Luminal	0.99	1.00e+00	[0.02, 5.60]	1	435	289	124370
RNAz	BL > Luminal	3.01	8.19e-02	[0.61, 9.00]	3	773	160	124032
	BL < Luminal	1.11	7.02e-01	[0.13, 4.10]	2	773	288	124032
miRNAs	BL > Luminal	0.00	1.00e+00	[0.00,12.20]	0	236	163	124569
	BL < Luminal	0.00	1.00e+00	[0.00, 6.80]	0	236	290	124569
snoRNAs or scaRNAs	BL > Luminal	7.09	9.63e-03	[1.44,21.30]	3	329	160	124476
	BL < Luminal	2.63	1.79e-01	[0.32, 9.70]	2	329	288	124476
SISSIz	BL > Luminal	0.00	1.92e-01	[0.00, 1.40]	0	1976	163	122829
	BL < Luminal	1.99	5.32e-02	[0.90, 3.80]	9	1976	281	122829

Annotation	Survey	Fisher's exact test: Odds ratio	P-value	95% CI	overlap: DE-Probes	BG	no overlap: DE-Probes	BG
TINs	BL > Luminal	3.54	8.60e-04	[1.66, 6.70]	10	2260	153	122545
	BL < Luminal	0.38	1.86e-01	[0.05, 1.40]	2	2260	288	122545
TUCP	BL > Luminal	4.28	8.19e-02	[0.51,15.80]	2	361	161	124444
	BL < Luminal	1.19	5.69e-01	[0.03, 6.70]	1	361	289	124444
Overlap with known non-coding RNAs regulated during cell cycle, upon TP53 or Stat3 induction (non-coding DE-probes)								
Normal vs. Tumor								
CC (intergenic)	Normal > Tumor	2.75	7.14e-06	[1.79, 4.06]	27	437	2797	124368
	Normal < Tumor	0.59	8.28e-02	[0.30, 1.05]	12	437	5754	124368
CC (intron)	Normal > Tumor	2.15	1.13e-12	[1.76, 2.60]	116	2440	2708	122365
	Normal < Tumor	0.63	4.45e-05	[0.49, 0.79]	71	2440	5695	122365
P53 (intergenic)	Normal > Tumor	3.33	7.85e-12	[2.42, 4.48]	48	645	2776	124160
	Normal < Tumor	1.01	9.25e-01	[0.67, 1.45]	30	645	5736	124160
P53 (intron)	Normal > Tumor	2.41	5.33e-40	[2.14, 2.72]	328	6445	2496	118360
	Normal < Tumor	0.52	2.36e-18	[0.44, 0.61]	160	6445	5606	118360
Stat3 (intergenic)	Normal > Tumor	0.50	2.98e-01	[0.10, 1.47]	3	266	2821	124539
	Normal < Tumor	0.49	7.62e-02	[0.18, 1.08]	6	266	5760	124539
Stat3 (intron)	Normal > Tumor	0.67	3.97e-01	[0.24, 1.47]	6	397	2818	124408
	Normal < Tumor	0.98	1.00e+00	[0.58, 1.57]	18	397	5748	124408
Basal-like vs. Luminal Tumors								
CC (intergenic)	BL > Luminal	7.16	2.79e-03	[1.92,18.80]	4	437	159	124368
	BL < Luminal	0.98	1.00e+00	[0.03, 5.60]	1	437	289	124368
CC (intron)	BL > Luminal	1.92	1.42e-01	[0.69, 4.30]	6	2440	157	122365
	BL < Luminal	1.42	2.87e-01	[0.61, 2.80]	8	2440	282	122365
P53 (intergenic)	BL > Luminal	11.25	2.44e-07	[5.03,22.00]	9	645	154	124160
	BL < Luminal	0.67	1.00e+00	[0.02, 3.80]	1	645	289	124160
P53 (intron)	BL > Luminal	3.33	1.19e-06	[2.08, 5.10]	25	6445	138	118360
	BL < Luminal	1.22	4.23e-01	[0.71, 2.00]	18	6445	272	118360
Stat3 (intergenic)	BL > Luminal	0.00	1.00e+00	[0.00,10.80]	0	266	163	124539
	BL < Luminal	1.62	4.62e-01	[0.04, 9.20]	1	266	289	124539
Stat3 (intron)	BL > Luminal	0.00	1.00e+00	[0.00, 7.20]	0	397	163	124408
	BL < Luminal	2.18	2.37e-01	[0.26, 8.00]	2	397	288	124408
Overlap with regulation tracks (non-coding DE-probes)								
Normal vs. Tumor								
CpG	Normal > Tumor	2.06	6.71e-08	[1.60, 2.62]	72	1565	2752	123240
	Normal < Tumor	2.92	5.06e-36	[2.50, 3.38]	206	1565	5560	123240
CpG and H3K4me3	Normal > Tumor	2.17	2.99e-08	[1.67, 2.78]	68	1402	2756	123403
	Normal < Tumor	2.95	1.82e-33	[2.51, 3.45]	187	1402	5579	123403
DNaseI	Normal > Tumor	1.51	2.68e-24	[1.39, 1.63]	1041	34856	1783	89949
	Normal < Tumor	1.34	7.21e-24	[1.26, 1.42]	1969	34856	3797	89949
H3K27ac	Normal > Tumor	2.81	7.82e-160	[2.61, 3.04]	1613	40108	1211	84697
	Normal < Tumor	1.34	9.55e-26	[1.27, 1.42]	2240	40108	3526	84697
H3k27me3	Normal > Tumor	0.51	1.26e-63	[0.48, 0.56]	1616	90135	1208	34670
	Normal < Tumor	1.34	1.63e-20	[1.26, 1.43]	4480	90135	1286	34670

Annotation	Survey	Fisher's exact test: Odds ratio	P-value	95% CI	overlap: DE-Probes	BG	no overlap: DE-Probes	BG
H3K36me3	Normal > Tumor	4.86	1.40e-243	[4.35, 5.44]	2458	72412	366	52393
	Normal < Tumor	1.01	8.06e-01	[0.95, 1.06]	3355	72412	2411	52393
H3K4me1	Normal > Tumor	2.43	6.65e-89	[2.21, 2.68]	2281	79050	543	45755
	Normal < Tumor	1.48	5.28e-41	[1.39, 1.57]	4144	79050	1622	45755
H3K4me3	Normal > Tumor	2.54	2.48e-124	[2.36, 2.74]	1304	31493	1520	93312
	Normal < Tumor	1.62	3.04e-62	[1.53, 1.71]	2039	31493	3727	93312
Pol II	Normal > Tumor	4.15	2.04e-279	[3.84, 4.47]	1389	23618	1435	101187
	Normal < Tumor	1.41	1.26e-26	[1.33, 1.50]	1428	23618	4338	101187
TFBs (Encode)	Normal > Tumor	1.76	1.82e-39	[1.62, 1.91]	869	25175	1955	99630
	Normal < Tumor	1.53	9.03e-43	[1.44, 1.63]	1609	25175	4157	99630
Basal-like vs. Luminal Tumors								
CpG	BL > Luminal	1.48	4.63e-01	[0.30, 4.40]	3	1565	160	123240
	BL < Luminal	2.23	3.17e-02	[0.95, 4.47]	8	1565	282	123240
CpG and H3K4me3	BL > Luminal	1.65	4.36e-01	[0.34, 4.92]	3	1402	160	123403
	BL < Luminal	2.18	4.76e-02	[0.87, 4.56]	7	1402	283	123403
DNaseI	BL > Luminal	1.24	1.91e-01	[0.88, 1.74]	53	34856	110	89949
	BL < Luminal	1.42	4.80e-03	[1.11, 1.82]	103	34856	187	89949
H3K27ac	BL > Luminal	2.74	2.12e-10	[1.99, 3.78]	92	40108	71	84697
	BL < Luminal	1.29	3.77e-02	[1.01, 1.65]	110	40108	180	84697
H3k27me3	BL > Luminal	0.34	7.63e-12	[0.24, 0.46]	76	90135	87	34670
	BL < Luminal	1.26	1.01e-01	[0.95, 1.67]	222	90135	68	34670
H3K36me3	BL > Luminal	10.00	5.48e-24	[5.43,20.45]	152	72412	11	52393
	BL < Luminal	1.07	5.92e-01	[0.84, 1.36]	173	72412	117	52393
H3K4me1	BL > Luminal	2.37	2.88e-06	[1.60, 3.61]	131	79050	32	45755
	BL < Luminal	1.13	3.29e-01	[0.88, 1.46]	192	79050	98	45755
H3K4me3	BL > Luminal	1.82	3.90e-04	[1.30, 2.52]	62	31493	101	93312
	BL < Luminal	1.23	1.19e-01	[0.94, 1.59]	85	31493	205	93312
Pol II	BL > Luminal	4.23	9.39e-19	[3.07, 5.83]	81	23618	82	101187
	BL < Luminal	1.41	1.31e-02	[1.07, 1.86]	72	23618	218	101187
TFBs (Encode)	BL > Luminal	1.33	1.18e-01	[0.91, 1.91]	41	25175	122	99630
	BL < Luminal	1.75	2.61e-05	[1.35, 2.26]	89	25175	201	99630
