# Supplementary material for: Long Non-Coding RNAs Differentially Expressed between Normal versus Primary Breast Tumor Tissues Disclose Converse Changes to Breast Cancer-Related Protein-Coding Genes
Source: PLoS One. 2014 Sep 29;9(9):e106076. doi: 10.1371/journal.pone.0106076 (PMC4180073; doi:10.1371/journal.pone.0106076)
Supplement: Table S6 — KEGG pathway enrichment analysis for mRNAs with intergenic, intronic, or antisense non-coding DE-probes. Most enriched KEGG pathways () of significantly differentially expressed protein-coding genes (Gencode release v12, ) with a non-coding DE-probe () either located in intergenic space and proximal to the protein-coding gene, located in intron of the protein-coding gene, or antisense to the protein-coding gene. Column headings indicate ID of KEGG pathway (ID), significance of enrichment (P-value), odds ratios (Odds ratio), expected number of genes associated with tested pathway (Exp. count), number of significantly differentially expressed genes associated with this pathway (Count), number of genes from the gene universe that are annotated in that pathway (Size), name of the pathway (Pathway Name), and a list of genes which are regulated in that pathway and significantly differentially expressed. Analysis was done by using the Bioconductor GOstats package. Mapping of genes to Entrez IDs is based on the NCBI gene information table (version: July 1, 2012). Significance of enrichment was assessed by a one-sided hypergeometric test where the universe contains all genes of the custom microarray which passed unspecific filtering (see Materials and Methods). (PDF) [file pone.0106076.s013.pdf]

	ID	P-value	Odds ratio	Exp. count	Count	Size	Pathway Name	Genes
Non-coding DE-probe in intergenic space but proximal to protein-coding gene								
1	05200	1.098E-05	3.086	10.048	25	217	Pathways in cancer	AR, BCL2, RUNX1T1, CBLB, CTNNB1, ETS1, FGFR1, FOS, FZD2, GLI3, IGF1, ITGB1, KIT, MET, MMP9, PIK3R1, RARA, STAT3, TCF7, TCF7L2, WNT5A, ZBTB16, FZD4, FZD7, SUFU
2	05217	1.110E-05	8.228	1.528	9	33	Basal cell carcinoma	CTNNB1, FZD2, GLI3, TCF7, TCF7L2, WNT5A, FZD4, FZD7, SUFU
3	04916	2.435E-05	5.138	2.963	12	64	Melanogenesis	CALM1, CTNNB1, EDN1, EDNRB, FZD2, GNAI2, KIT, TCF7, TCF7L2, WNT5A, FZD4, FZD7
4	05221	4.461E-04	5.257	1.898	8	41	Acute myeloid leukemia	RUNX1T1, KIT, PIK3R1, RARA, STAT3, TCF7, TCF7L2, ZBTB16
5	04520	1.002E-03	4.557	2.130	8	46	Adherens junction	CTNNB1, FGFR1, MET, MLLT4, PVRL1, SRC, TCF7, TCF7L2
6	04360	1.272E-03	3.347	3.843	11	83	Axon guidance	EFNA5, EFNB2, EPHA4, GNAI2, ITGB1, MET, CXCL12, SEMA5A, ROBO3, UNC5A, SEMA3D
7	05412	3.094E-03	4.177	1.991	7	43	Arrhythmogenic right ventricular cardiomyopathy (ARVC)	CTNNB1, ITGA5, ITGB1, SGCA, TCF7, TCF7L2, CACNG4
8	05100	4.578E-03	3.852	2.130	7	46	Bacterial invasion of epithelial cells	CBLB, CTNNB1, ITGA5, ITGB1, MET, PIK3R1, SRC
9	04670	4.609E-03	3.447	2.686	8	58	Leukocyte transendothelial migration	CTNNB1, GNAI2, ITGB1, MLLT4, MMP9, PIK3R1, MAPK13, CXCL12
10	05215	6.301E-03	3.248	2.824	8	61	Prostate cancer	AR, BCL2, CTNNB1, FGFR1, IGF1, PIK3R1, TCF7, TCF7L2
11	04950	2.426E-02	5.727	0.648	3	14	Maturity onset diabetes of the young	FOXA2, FOXA3, PAX4
12	05410	2.613E-02	2.897	2.315	6	50	Hypertrophic cardiomyopathy (HCM)	IGF1, ITGA5, ITGB1, SGCA, TPM1, CACNG4
13	05210	2.613E-02	2.897	2.315	6	50	Colorectal cancer	BCL2, CTNNB1, FOS, PIK3R1, TCF7, TCF7L2
14	05414	3.106E-02	2.769	2.408	6	52	Dilated cardiomyopathy	IGF1, ITGA5, ITGB1, SGCA, TPM1, CACNG4

	ID	P-value	Odds ratio	Exp. count	Count	Size	Pathway Name	Genes
15	04971	3.201E-02	3.111	1.806	5	39	Gastric acid secretion	CALM1, GNAI2, ITPR3, KCNJ2, KCNJ16
16	04340	3.791E-02	3.511	1.296	4	28	Hedgehog signaling pathway	GAS1, GLI3, WNT5A, SUFU
17	04630	3.830E-02	2.402	3.195	7	69	Jak-STAT signaling pathway	CBLB, PIK3R1, STAT3, SOCS3, SPRY1, SPRY2, SPRY4
Non-coding DE-probe in intron of protein-coding gene								
1	04520	1.783E-06	6.042	3.286	14	46	Adherens junction	CTNND1, IGF1R, PTPRF, PTPRJ, PTPRM, PVRL1, TCF7, TCF7L2, TGFBR1, VCL, IQGAP1, SORBS1, PARD3, PVRL4
2	04510	1.418E-04	2.817	8.929	21	125	Focal adhesion	BCL2, CAV1, CCND3, COL4A1, COL6A1, COL6A2, FLNC, IGF1R, ITGA9, ITGB8, LAMA3, LAMB3, LAMC1, MYLK, PXN, TNR, VASP, VCL, VEGFC, SHC2, PDGFC
3	04144	3.735E-04	2.739	8.214	19	115	Endocytosis	CAV1, DAB2, DNM1, DNM2, IGF1R, LDLR, MDM2, PSD, RAB5C, TGFBR1, IQSEC1, VPS4A, SH3KBP1, PARD3, SH3GLB2, VPS25, RAB11FIP4, FAM125B, ARAP1
4	05412	6.566E-04	4.095	3.071	10	43	Arrhythmogenic right ventricular cardiomyopathy (ARVC)	CACNA1C, DMD, ITGA9, ITGB8, JUP, LMNA, SGCD, TCF7, TCF7L2, CACNA2D2
5	04512	2.642E-03	3.286	3.643	10	51	ECM-receptor interaction	COL4A1, COL6A1, COL6A2, ITGA9, ITGB8, LAMA3, LAMB3, LAMC1, TNR, AGRN
6	04974	7.225E-03	3.242	2.929	8	41	Protein digestion and absorption	ATP1A1, COL4A1, COL6A1, COL6A2, ELN, MME, SLC7A8, COL18A1
7	05217	7.378E-03	3.591	2.357	7	33	Basal cell carcinoma	GLI3, TCF7, TCF7L2, TP53, AXIN2, SUFU, WNT5B
8	04722	7.752E-03	2.374	6.214	13	87	Neurotrophin signaling pathway	BCL2, CAMK2B, CAMK2G, GAB1, NGFR, NTF3, NTRK2, NTRK3, RPS6KA2, TP53, MAPKAPK2, SH2B3, SHC2
9	05215	9.955E-03	2.631	4.357	10	61	Prostate cancer	AR, BCL2, FOXO1, IGF1R, MDM2, TCF7, TCF7L2, TP53, CREB5, PDGFC
10	05414	1.003E-02	2.802	3.714	9	52	Dilated cardiomyopathy	ADCY6, CACNA1C, DMD, ITGA9, ITGB8, LMNA, SGCD, TPM1, CACNA2D2
11	04360	1.340E-02	2.274	5.929	12	83	Axon guidance	EFNA5, EPHB1, GNAI2, ABLIM1, LIMK2, RGS3, SLIT1, SLIT3, SEMA5A, SEMA6D, UNC5B, SEMA3D

	ID	P-value	Odds ratio	Exp. count	Count	Size	Pathway Name	Genes
12	05200	1.810E-02	1.701	15.500	24	217	Pathways in cancer	FAS, AR, BCL2, BCL2L1, COL4A1, FOXO1, FOS, GLI3, IGF1R, JUP, LAMA3, LAMB3, LAMC1, MDM2, RARA, TCF7, TCF7L2, TGFBR1, TP53, VEGFC, ZBTB16, AXIN2, SUFU, WNT5B
13	04971	1.845E-02	2.911	2.786	7	39	Gastric acid secretion	ADCY6, ATP1A1, CAMK2B, CAMK2G, GNAI2, KCNJ2, MYLK
14	05410	2.337E-02	2.538	3.571	8	50	Hypertrophic cardiomyopathy (HCM)	CACNA1C, DMD, ITGA9, ITGB8, LMNA, SGCD, TPM1, CACNA2D2
15	05100	4.213E-02	2.382	3.286	7	46	Bacterial invasion of epithelial cells	CAV1, DNM1, DNM2, GAB1, PXN, VCL, SHC2
Non-coding DE-probe antisense to protein-coding gene								
1	04520	1.509E-03	3.605	3.401	10	46	Adherens junction	CSNK2A1, CTNNB1, EP300, IGF1R, PTPN1, SRC, TCF7L2, TGFBR1, TGFBR2, VCL
2	05100	1.509E-03	3.605	3.401	10	46	Bacterial invasion of epithelial cells	CAV1, CAV2, CLTC, CRK, CTNNB1, DNM1, GAB1, PXN, SRC, VCL
3	05215	4.293E-03	2.854	4.510	11	61	Prostate cancer	AR, BCL2, CDK2, CDKN1A, CTNNB1, E2F1, EP300, IGF1R, PDGFRB, TCF7L2, CREB3L2
4	04110	6.922E-03	2.415	6.137	13	83	Cell cycle	BUB1B, CDC25A, CDC25B, CDK2, CDKN1A, E2F1, EP300, MCM2, MCM3, PCNA, PLK1, SMC1A, BUB3
5	05200	1.482E-02	1.718	16.045	25	217	Pathways in cancer	BIRC5, AR, BCL2, BCL2L1, BRCA2, RUNX1, CDK2, CDKN1A, CRK, CTNNB1, E2F1, EP300, ETS1, FGF1, FOS, FZD2, IGF1R, ITGA2, PDGFRB, RARA, STAT1, TCF7L2, TGFBR1, TGFBR2, FZD5
6	03030	1.611E-02	3.986	1.553	5	21	DNA replication	MCM2, MCM3, PCNA, RPA1, POLD3
7	04350	1.992E-02	2.632	3.475	8	47	TGF-beta signaling pathway	BMP6, BMPR1B, DCN, EP300, ID1, INHBA, TGFBR1, TGFBR2
8	04144	2.024E-02	1.948	8.503	15	115	Endocytosis	CAV1, CAV2, CLTC, DNM1, IGF1R, LDLR, PSD, SRC, TFRC, TGFBR1, TGFBR2, RAB31, GIT1, ASAP1, RAB11FIP4
9	03430	2.607E-02	4.238	1.183	4	16	Mismatch repair	PCNA, RPA1, EXO1, POLD3

	ID	P-value	Odds ratio	Exp. count	Count	Size	Pathway Name	Genes
10	04916	4.326E-02	2.097	4.732	9	64	Melanogenesis	CALM2, CAMK2B, CTNNB1, EP300, FZD2, GNAS, TCF7L2, FZD5, CREB3L2
11	05220	4.651E-02	2.178	4.067	8	55	Chronic myeloid leukemia	BCL2L1, RUNX1, CDKN1A, CRK, E2F1, TGFBR1, TGFBR2, GAB2
