# Supplementary material for: Long Non-Coding RNAs Differentially Expressed between Normal versus Primary Breast Tumor Tissues Disclose Converse Changes to Breast Cancer-Related Protein-Coding Genes
Source: PLoS One. 2014 Sep 29;9(9):e106076. doi: 10.1371/journal.pone.0106076 (PMC4180073; doi:10.1371/journal.pone.0106076)
Supplement: Table S7 — Detailed documentation of used annotation categories. Column headings Annotation, Abbreviation, Source/URL, Assembly, Citation, and Comment indicate the according genomic feature, the abbreviation used in figures and tables throughout the paper, the online source of the annotation data set, the human genome assembly for which annotation was available, references, and comments about preprocessing of the annotation data, respectively. (PDF) [file pone.0106076.s014.pdf]

Annotation	Abbreviation	Source/URL	Assembly	Citation	Comment
Protein-coding gene annotation					
Coding exons	CDS	Gencode v12	GRCh37/hg19	Gencode [1]	-
Introns	intron	Gencode v12 and UCSC table browser (tracks: UCSC genes, RefSeq genes, Ensembl genes)	GRCh37/hg19	Gencode [1], Ensembl [2], RefSeq [3], UCSC [4]; [5]	Defined as intronic nucleotides which do not overlap any exon of a protein-coding transcript.
Intergenic	intergenic	Gencode v12 and UCSC table browser (tracks: UCSC genes, RefSeq genes, Ensembl genes)	GRCh37/hg19	Gencode [1], Ensembl [2], RefSeq [3], UCSC [4]; [5]	Defined as the complement of all known protein-coding transcripts.
UTRs	UTRs	Gencode v12	GRCh37/hg19	Gencode [1]	-
Non-coding gene annotation					
Long non-coding RNAs	lncRNAs (Gencode)	ftp://ftp.sanger.ac.uk/pub/gencode/release_12/gencode.v12.long_noncoding_RNAs.gtf.gz	GRCh37/hg19	Gencode [1]	The original set of long non-coding RNAs as annotated in Gencode was reduced to a set of *bona fide* non-coding RNAs without any evidence for functional short ORFs (see descriptions above).
Large intergenic non-coding RNAs	lincRNAs	http://www.broadinstitute.org/genome_bio/human_lincrnas/sites/default/files/lincRNA_catalog/lincRNAs_transcripts.bed	GRCh37/hg19	[6]	-
Transcripts of uncertain coding potential	TUCP	http://www.broadinstitute.org/genome_bio/human_lincrnas/sites/default/files/TUCP_transcripts_catalog/TUCP_transcripts.gtf	GRCh37/hg19	[6]	-
Chromatin associated RNAs	CARs	-	NCBI36/hg18	[7]	Mapped to GRCh37/hg19 using liftOver [5].

Annotation	Abbreviation	Source/URL	Assembly	Citation	Comment
LncRNAdb	lncRNAdb	http://lncrnadb.com	-	[8]	Coordinates in GRCh37/hg19 have been derived by BLAT [9] with parameters -trimHardA -minIdentity=95.
Short RNAs	miRNAs, snoRNAs, scaRNAs	UCSC table browser (track: sno/miRNA)	GRCh37/hg19	[10, 11, 5]	-
Intronic non-coding RNAs	TINs, PINs	UCSC Genome Browser mirror for functional RNA (http://www.ncrna.org/glocal/cgi-bin/hgGateway)	NCBI36/hg18	[12]	Mapped to GRCh37/hg19 using liftOver [5]. The original set of human intronic non-coding RNAs [12] was reassessed according to gene annotation in hg19 (see descriptions above).
Regions of conserved secondary structure					
RNAz	RNAz	-	GRCh37/hg19	[13]	-
SISSIz	SISSIz	-	GRCh37/hg19	[13]	-
Evofold	Evofold	UCSC table browser (track: EvoFold)	GRCh37/hg19	[14, 5]	-
Regulation Tracks					
H3K4 trimethylation	H3K4me3	ftp://hgdownload.cse.ucsc.edu/goldenPath/hg18/encodeDCC/wgEncodeBroadChipSeq/	NCBI36/hg18	[15, 5]	Chromatin-mark associated with promoter sites [16, 17]. Mapped to GRCh37/hg19 using liftOver [5].
CpG islands	CpG	UCSC table browser (track: CpG Islands)	GRCh37/hg19	[18, 5]	Associated with transcription start sites [19, 20].
DNaseI-hypersensitive sites	DNaseI	UCSC table browser (track: DNaseI Clusters)	GRCh37/hg19	[15, 5]	Associated with transcription factor binding sites [17, 21].
Transcription factor binding sites (TFBs)	TFBs (Encode)	UCSC table browser (track: Txn Factor ChIP)	GRCh37/hg19	[15, 5]	Binding sites identified by ChIP-seq [15].
PolII binding sites	POL-II	ftp://hgdownload.cse.ucsc.edu/goldenPath/hg18/encodeDCC/wgEncodeBroadChipSeq/	NCBI36/hg18	[15, 5]	PolII binding sites derived by ChIP-seq [15]. Mapped to GRCh37/hg19 using liftOver [5].

Annotation	Abbreviation	Source/URL	Assembly	Citation	Comment
H3K36 trimethylation	H3K36me3	`ftp://hgdownload.cse.ucsc.edu/goldenPath/hg18/encodeDCC/wgEncodeBroadChipSeq/`	NCBI36/hg18	[15, 5]	Chromatin-mark associated with active regions of PolII transcripts [22]. Mapped to GRCh37/hg19 using `liftOver` [5].
H3K27 trimethylation	H3K27me3	`ftp://hgdownload.cse.ucsc.edu/goldenPath/hg18/encodeDCC/wgEncodeBroadChipSeq/`	NCBI36/hg18	[15, 5]	Chromatin-mark associated with repressed regions of PolII transcripts [23]. Mapped to GRCh37/hg19 using `liftOver` [5].
H3K4 monomethylation	H3K4me1	`ftp://hgdownload.cse.ucsc.edu/goldenPath/hg18/encodeDCC/wgEncodeBroadChipSeq/`	NCBI36/hg18	[15, 5]	Chromatin-mark associated with enhancer regions [24, 17]. Mapped to GRCh37/hg19 using `liftOver` [5].
H3K27 acetylation	H3K27ac	`ftp://hgdownload.cse.ucsc.edu/goldenPath/hg18/encodeDCC/wgEncodeBroadChipSeq/`	NCBI36/hg18	[15, 5]	Chromatin-mark associated with enhancer and promoter sites [15, 25, 26].Mapped to GRCh37/hg19 using `liftOver` [5].
Other					
Repeats	-	`UCSC` table browser (track: RepeatMasker)	GRCh37/hg19	[27, 5]	-
Genome gaps	-	`UCSC` table browser (track: Gap)	GRCh37/hg19	[5]	-

References

[1] Harrow J, Frankish A, Gonzalez JM, Tapanari E, Diekhans M, et al. (2012) GENCODE: the reference human genome annotation for The ENCODE Project. Genome Res 22: 1760–1774.

[2] Hubbard T, Barker D, Birney E, Cameron G, Chen Y, et al. (2002) The Ensembl genome database project. Nucleic Acids Res 30: 38–41.

[3] Pruitt KD, Tatusova T, Maglott DR (2007) NCBI reference sequences (RefSeq): a curated non-redundant sequence database of genomes, transcripts and proteins. Nucleic Acids Res 35: D61–D65.

[4] Meyer LR, Zweig AS, Hinrichs AS, Karolchik D, Kuhn RM, et al. (2013) The UCSC genome browser database: extensions and updates 2013. Nucleic Acids Res : D64-9.

[5] Kuhn RM, Haussler D, Kent WJ (2012) The UCSC genome browser and associated tools. Brief Bioinformatics .

[6] Cabili MN, Trapnell C, Goff L, Koziol M, Tazon-Vega B, et al. (2011) Integrative annotation of human large intergenic noncoding RNAs reveals global properties and specific subclasses. Genes Dev 25: 1915–1927.

[7] Mondal T, Rasmussen M, Pandey GK, Isaksson A, Kanduri C (2010) Characterization of the RNA content of chromatin. Genome Res 20: 899–907.

[8] Amaral P, Clark M, Gascoigne D, Dinger M, Mattick J (2011) lncRNAdb: a reference database for long noncoding RNAs. Nucleic Acids Res 39: D146–D151.

[9] Kent WJ (2002) BLAT–the BLAST-like alignment tool. Genome Res 12: 656–664.

[10] Lestrade L, Weber MJ (2006) snoRNA-LBME-db, a comprehensive database of human H/ACA and C/D box snoRNAs. Nucleic Acids Res 34: D158–D162.

[11] Griffiths-Jones S (2004) The microRNA Registry. Nucleic Acids Res 32: D109–D111.

[12] Nakaya HI, Amaral PP, Louro R, Lopes A, Fachel AA, et al. (2007) Genome mapping and expression analyses of human intronic noncoding RNAs reveal tissue-specific patterns and enrichment in genes related to regulation of transcription. Genome Biol 8: R43.

[13] Smith MA, Gesell T, Stadler PF, Mattick JS (2013) Widespread purifying selection on RNA structure in mammals. Nucleic Acids Res 41: 8220-8236.

[14] Pedersen JS, Bejerano G, Siepel A, Rosenbloom K, Lindblad-Toh K, et al. (2006) Identification and classification of conserved RNA secondary structures in the human genome. PLoS Comput Biol 2: e33.

[15] Birney E, Stamatoyannopoulos J, Dutta A, Guigó R, Gingeras T, et al. (2007) Identification and analysis of functional elements in 1% of the human genome by the ENCODE pilot project. Nature 447: 799–816.

[16] Roh TY, Cuddapah S, Cui K, Zhao K (2006) The genomic landscape of histone modifications in human T cells. Proc Natl Acad Sci U S A 103: 15782–15787.

[17] Bulger M, Groudine M (2010) Enhancers: the abundance and function of regulatory sequences beyond promoters. Dev Biol 339: 250–257.

[18] Gardiner-Garden M, Frommer M (1987) CpG islands in vertebrate genomes. J Mol Biol 196: 261–282.

[19] Deaton AM, Bird A (2011) CpG islands and the regulation of transcription. Genes Dev 25: 1010–1022.

[20] Guenther MG, Levine SS, Boyer LA, Jaenisch R, Young RA (2007) A chromatin landmark and transcription initiation at most promoters in human cells. Cell 130: 77–88.

[21] Xi H, Shulha HP, Lin JM, Vales TR, Fu Y, et al. (2007) Identification and characterization of cell type-specific and ubiquitous chromatin regulatory structures in the human genome. PLoS Genet 3: e136.

[22] Mikkelsen TS, Ku M, Jaffe DB, Issac B, Lieberman E, et al. (2007) Genome-wide maps of chromatin state in pluripotent and lineage-committed cells. Nature 448: 553–560.

[23] Barski A, Cuddapah S, Cui K, Roh TY, Schones DE, et al. (2007) High-resolution profiling of histone methylations in the human genome. Cell 129: 823–837.

[24] Heintzman ND, Stuart RK, Hon G, Fu Y, Ching CW, et al. (2007) Distinct and predictive chromatin signatures of transcriptional promoters and enhancers in the human genome. Nat Genet 39: 311–318.

[25] Terrenoire E, McRonald F, Halsall JA, Page P, Illingworth RS, et al. (2010) Immunostaining of modified histones defines high-level features of the human metaphase epigenome. Genome Biol 11: R110.

[26] Shin JH, Li RW, Gao Y, Baldwin R 6th, Li Cj (2012) Genome-wide ChIP-seq mapping and analysis reveal butyrate-induced acetylation of H3K9 and H3K27 correlated with transcription activity in bovine cells. Funct Integr Genomics 12: 119–130.

[27] Smit A, Hubley R, Green P (2010) RepeatMasker Open-3.0. URL `http://www.repeatmasker.org`.
