# Supplementary material for: Long Non-Coding RNAs Differentially Expressed between Normal versus Primary Breast Tumor Tissues Disclose Converse Changes to Breast Cancer-Related Protein-Coding Genes
Source: PLoS One. 2014 Sep 29;9(9):e106076. doi: 10.1371/journal.pone.0106076 (PMC4180073; doi:10.1371/journal.pone.0106076)
Supplement: Table S8 — RT-qPCR primers. LncRNA expression was validated by RT-qPCR with primers designed using Primer3 (v0.4.0) with default parameters. (PDF) [file pone.0106076.s015.pdf]

	RT-qPCR primers (from $5'$ to $3'$ end)	
Transcript ID	forward	reverse
CAR-FTX	CCC ATC CAG CAG GGA TAC TA	CAG AAT TGC TTA ATT GTG GCA TTA
CAR-CALD1	GAA ACT TTT GGC ACA GGC TTA	AGA GGG AGC AAA CAG CTT CA
CAR-HNRNPH1	GAG GGC AAT CCT CAA TGA AA	GGC TAG CTT ATG GCA AGG TG
HDAC3 mRNA	TGA GAC ATC GCT GCT GGT AG	GGT CCA GAT ACT GGC GTG AG
HDAC3 antisense RNA	AGA GCC CCT TCC AAA TCT CT	CAA GGA CTG AGA TTG CCT CTG
β-Actin mRNA	CCT GGC ACC CAG CAC AAT	GCC GAT CCA CAC GGA GTA CT
