# Supplementary material for: Long Non-Coding RNAs Differentially Expressed between Normal versus Primary Breast Tumor Tissues Disclose Converse Changes to Breast Cancer-Related Protein-Coding Genes
Source: PLoS One. 2014 Sep 29;9(9):e106076. doi: 10.1371/journal.pone.0106076 (PMC4180073; doi:10.1371/journal.pone.0106076)
Supplement: Methods S1 — Description of genomic annotation categories. Detailed description of all genomic annotation categories used to investigate the genomic location of DE-probes. (PDF) [file pone.0106076.s016.pdf]

1 Supplemental Methods S1

1.1 Annotation categories

For a complete annotation of known protein-coding genes we relied on RefSeq [1], UCSC [2], Ensembl [3], and Gencode v12 [4]. The first three datasets were downloaded from the University of California Santa Cruz (UCSC) table browser (hg19), while Gencode annotation was directly taken from http://www.gencodegenes.org/releases/12.html. For all sets the genomic coordinates of protein-coding genes, protein-coding transcript isoforms, and protein-coding exons (CDS) were used to define the coordinates of known protein-coding genes, transcript variants and exons, respectively. Intronic regions were defined by intervals annotated as an intron in at least one of the gene annotations sets above, but never annotated as an exon of a protein-coding transcript. Intergenic regions were defined as the complement of all protein-coding transcript variants known in at least one of the above annotation sets. For untranslated regions (UTRs) and pseudogenes we relied solely on the coordinates as defined in Gencode v12.

Annotation for known non-coding RNA genes has been collected from different sites: (1) A set of *bona fide* intergenic long non-coding RNAs was constructed from the 18,855 transcripts defined in the long non-coding RNA dataset of Gencode v12. In order to exclude non-coding isoforms of protein-coding genes and antisense RNAs, we discarded all those transcripts that overlapped at least one known protein-coding transcript, no matter of reading direction (Gencode v12 - 7,401 transcripts; UCSC, Ensembl, and RefSeq protein-coding genes - 8,671). To further exclude transcripts predicted to contain conserved short open reading frames, we discarded all those transcripts with an exon that overlapped a significant RNAcode [5] segment (p-value<0.05, 7,500 transcripts), or if not scored by RNAcode, an exon that overlaps a significant tblastn hit (E-value<0.05, RefSeq database from March 7, 2012; 8,848 transcripts). The filtering steps resulted in 5,209 long non-coding transcripts which corresponded to 3,814 non-coding genes. (2) Large intergenic non-coding RNAs (lincRNAs) and transcripts of uncertain coding potential (TUCPs) as detected in a comprehensive expression study across 22 human tissues and cell lines have been downloaded from the Human Body Map catalog (http://www.broadinstitute.org/genome_bio/human_lincrnas/) [6]. (3) Genomic coordinates of large RNAs found in chromatin were taken from [7]. (4) Sequences of validated large non-coding RNAs were downloaded from the lncRNAdb database [8] and mapped to the human genome version hg19 by employing BLAT [9] with parameters -trimHardA -minIdentity=95. (5) Genomic coordinates of known short RNAs, like miRNAs and snoRNAs, were downloaded from the wgRNA track available from the UCSC table browser, and split in a subset containing the precursors of miRNAs and a subset of C/D box and H/ACA box snoRNAs as well as small Cajal body-specific RNAs (scaRNAs) [10, 11]. (6) Human intronic non-coding RNAs [12] were downloaded from the UCSC Genome Browser mirror for functional RNA (http://www.ncrna.org/glocal/cgi-bin/hgGateway) and mapped to hg19. Original sets of totally intronic non-coding RNAs (TINs) and partially intronic non-coding RNAs (PINs) were reannotated according to Gencode v12 gene annotation (no matter of reading direction) in order to receive reliable sets of intronic non-coding RNAs. 31,023 TINs out of 55,126 original TINs mapping to hg19 were completely found in introns and did not overlap with conserved open reading frames as detected by RNAcode (p-value<0.05), or did not exhibit sequence similarity to known human amino acid sequences (tblastn, RefSeq database from March 7, 2012, E-value<0.05) if RNAcode could not be applied due to low sequence conservation. 621 intronic non-coding RNAs classified as TINs in [12] overlapped Gencode

v12 exons and were assigned to the set of partially intronic non-coding RNAs (PINs). 6,268 PINs out of 12,589 PINs mapping to hg19 were partially found in introns and did not overlap with conserved short open reading frames detected in introns (RNAcode, p-value< 0.05). 141 intronic non-coding RNAs originally annotated as PINs did not overlap Gencode v12 exons and, hence, have been added to the set of totally intronic non-coding RNAs (TINs).

The number of DE-probes with conserved secondary structure was retrieved by mapping their coordinates to genomic regions known to contain conserved secondary structure elements (Evofold [13], RNAz 2.0 [14, 15], and SISSIz [16]). For RNAz and SISSIz we relied on high scoring predictions from *Smith et al.* [17].

We retrieved genomic coordinates of selected histone modifications from the Encode consortium [18] in order to assess independent evidence for transcription initiation and elongation (including data for 6 normal cell lines, 1 cancer, and 1 embryonic stem cell line). To detect differential expression of known promoter-sites we relied on the histone modification H3K4 trimethylation, which marks promoter regions of actively transcribed genes [19, 20]. This chromatin mark often co-occurs with CpG islands, which are also associated with transcription start sites [21, 22]. In addition DNaseI-hypersensitive sites define regions where the chromatin structure is changed in a way such that transcription factor binding is possible [20, 23]. The genomic coordinates of transcription factor binding sites (TFBs) corresponded to binding sites identified by ChIP-seq [18] or found to be conserved within human/mouse/rat alignments [24]. Pol II binding sites were also derived from Encode to assess the fraction of genomic loci possibly transcribed by Polymerase II. Loci actively transcribed by Pol II are marked by H3K36me3 [25], while transcriptional silenced loci carry H3k27me3 histone modifications[26]. In contrast, H3K4me1 is associated with enhancer regions, but not with transcription start sites [27, 20], and H3K27Ac is associated with enhancer and promoter sites [18, 28, 29].

We used the R library genomeIntervals [30] to revise and adapt all annotation sets. A detailed listing of annotations sets and their sources is provided in Supplemental Table S7.

References

[1] Pruitt KD, Tatusova T, Maglott DR (2007) NCBI reference sequences (RefSeq): a curated non-redundant sequence database of genomes, transcripts and proteins. Nucleic Acids Res 35: D61–D65.

[2] Meyer LR, Zweig AS, Hinrichs AS, Karolchik D, Kuhn RM, et al. (2013) The UCSC genome browser database: extensions and updates 2013. Nucleic Acids Res : D64-9.

[3] Hubbard T, Barker D, Birney E, Cameron G, Chen Y, et al. (2002) The Ensembl genome database project. Nucleic Acids Res 30: 38–41.

[4] Harrow J, Frankish A, Gonzalez JM, Tapanari E, Diekhans M, et al. (2012) GENCODE: the reference human genome annotation for The ENCODE Project. Genome Res 22: 1760–1774.

[5] Washietl S, Findeiss S, Müller S, Kalkhof S, von BM, et al. (2011) RNAcode: Robust discrimination of coding and noncoding regions in comparative sequence data. RNA 17: 578–594.

[6] Cabili MN, Trapnell C, Goff L, Koziol M, Tazon-Vega B, et al. (2011) Integrative annotation of human large intergenic noncoding RNAs reveals global properties and specific subclasses. Genes Dev 25: 1915–1927.

[7] Mondal T, Rasmussen M, Pandey GK, Isaksson A, Kanduri C (2010) Characterization of the RNA content of chromatin. Genome Res 20: 899–907.

[8] Amaral P, Clark M, Gascoigne D, Dinger M, Mattick J (2011) lncRNAdb: a reference database for long noncoding RNAs. Nucleic Acids Res 39: D146–D151.

[9] Kent WJ (2002) BLAT–the BLAST-like alignment tool. Genome Res 12: 656–664.

[10] Lestrade L, Weber MJ (2006) snoRNA-LBME-db, a comprehensive database of human H/ACA and C/D box snoRNAs. Nucleic Acids Res 34: D158–D162.

[11] Griffiths-Jones S (2004) The microRNA Registry. Nucleic Acids Res 32: D109–D111.

[12] Nakaya HI, Amaral PP, Louro R, Lopes A, Fachel AA, et al. (2007) Genome mapping and expression analyses of human intronic noncoding RNAs reveal tissue-specific patterns and enrichment in genes related to regulation of transcription. Genome Biol 8: R43.

[13] Pedersen JS, Bejerano G, Siepel A, Rosenbloom K, Lindblad-Toh K, et al. (2006) Identification and classification of conserved RNA secondary structures in the human genome. PLoS Comput Biol 2: e33.

[14] Washietl S, Hofacker IL, Lukasser M, Hüttenhofer A, Stadler PF (2005) Mapping of conserved RNA secondary structures predicts thousands of functional noncoding RNAs in the human genome. Nat Biotechnol 23: 1383–1390.

[15] Gruber AR, Findeiss S, Washietl S, Hofacker IL, Stadler PF (2010) RNAz 2.0: Improved noncoding RNA detection. Pac Symp Biocomput 15: 69–79.

[16] Gesell T, Washietl S (2008) Dinucleotide controlled null models for comparative RNA gene prediction. BMC Bioinformatics 9: 248.

[17] Smith MA, Gesell T, Stadler PF, Mattick JS (2013) Widespread purifying selection on RNA structure in mammals. Nucleic Acids Res 41: 8220-8236.

[18] Birney E, Stamatoyannopoulos J, Dutta A, Guigó R, Gingeras T, et al. (2007) Identification and analysis of functional elements in 1% of the human genome by the ENCODE pilot project. Nature 447: 799–816.

[19] Roh TY, Cuddapah S, Cui K, Zhao K (2006) The genomic landscape of histone modifications in human T cells. Proc Natl Acad Sci U S A 103: 15782–15787.

[20] Bulger M, Groudine M (2010) Enhancers: the abundance and function of regulatory sequences beyond promoters. Dev Biol 339: 250–257.

[21] Deaton AM, Bird A (2011) CpG islands and the regulation of transcription. Genes Dev 25: 1010–1022.

[22] Guenther MG, Levine SS, Boyer LA, Jaenisch R, Young RA (2007) A chromatin landmark and transcription initiation at most promoters in human cells. Cell 130: 77–88.

[23] Xi H, Shulha HP, Lin JM, Vales TR, Fu Y, et al. (2007) Identification and characterization of cell type-specific and ubiquitous chromatin regulatory structures in the human genome. PLoS Genet 3: e136.

[24] Wingender E, Chen X, Hehl R, Karas H, Liebich I, et al. (2000) TRANSFAC: an integrated system for gene expression regulation. Nucleic Acids Res 28: 316–319.

[25] Mikkelsen TS, Ku M, Jaffe DB, Issac B, Lieberman E, et al. (2007) Genome-wide maps of chromatin state in pluripotent and lineage-committed cells. Nature 448: 553–560.

[26] Barski A, Cuddapah S, Cui K, Roh TY, Schones DE, et al. (2007) High-resolution profiling of histone methylations in the human genome. Cell 129: 823–837.

[27] Heintzman ND, Stuart RK, Hon G, Fu Y, Ching CW, et al. (2007) Distinct and predictive chromatin signatures of transcriptional promoters and enhancers in the human genome. Nat Genet 39: 311–318.

[28] Terrenoire E, McRonald F, Halsall JA, Page P, Illingworth RS, et al. (2010) Immunostaining of modified histones defines high-level features of the human metaphase epigenome. Genome Biol 11: R110.

[29] Shin JH, Li RW, Gao Y, Baldwin R 6th, Li Cj (2012) Genome-wide ChIP-seq mapping and analysis reveal butyrate-induced acetylation of H3K9 and H3K27 correlated with transcription activity in bovine cells. Funct Integr Genomics 12: 119–130.

[30] Gagneur J, Toedling J, Bourgon R, Delhomme N (2012) genomeIntervals: Operations on genomic intervals. R package version 1.14.0.
